# Intermediate-Controlled Synthesis of Quasi-2D (PEA)_2_MA_4_Pb_5_I_16_ in the 20–30% Relative Humidity Glovebox Environment for Fabricating Perovskite Solar Cells with 1 Month Durability in the Air

**DOI:** 10.1021/acsomega.4c06621

**Published:** 2024-11-27

**Authors:** Yen-Shuo Chen, Min-Han Hsieh, Ching-Chang Lin, Yi-Cheng Huang, Shang-Yu Tsai, Fu-Hsiang Ko

**Affiliations:** † Department of Materials Science and Engineering, National Yang Ming Chiao Tung University, 1001 University Road, Hsinchu 30010, Taiwan; ‡ Department of General Systems Studies, Graduate School of Arts and Sciences, The University of Tokyo, 3-8-1 Komaba, Meguro-ku, Tokyo 153-8902, Japan

## Abstract

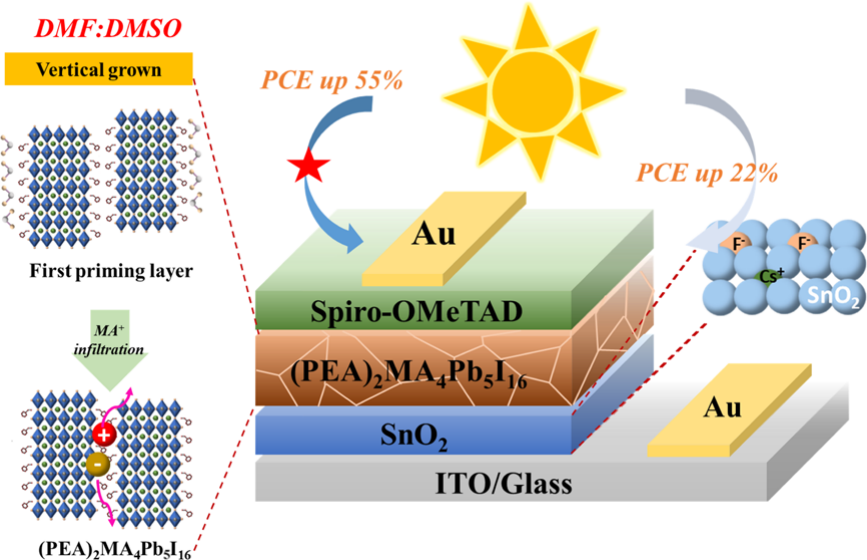

Herein, quasi-two-dimensional (Q-2D) (PEA)_2_MA_4_Pb_5_I_16_ (prepared by a two-step process) and hole transport layer of a solar cell were fabricated in a high relative humidity (25 ± 5%) environment. The PSC behavior of most Q-2D perovskites is worse than that of three-dimensional perovskites owing to the horizontal alignment of the innate characteristic organic plates on the substrate. Using hybrid immersion solvents (HISs), we have improved vertical alignment in an appropriate ratio to enhance the efficiency of charge transfer and the high coverage of the first priming layer (first step). The grazing incidence X-ray diffraction pattern of the optimized structures revealed a preferential orientation for the vertical alignment of (111), which improved the charge transfer in PSCs and micrometer-level grain size growth. The second step was processed in a high-humidity environment (50 ± 5%) (methylammonium iodide solution embedded), and Q-2D (PEA)_2_MA_4_Pb_5_I_16_ demonstrated distinct grain boundaries. The power conversion efficiency (PCE, 13.09%) of the champion device of the first priming layer prepared using the HIS system increased by >55% compared to the single-immersion solvent (8.3%). The PCE of the ion-modified ETL PSCs was 16.02% (CsF-3) and 14.58% (CsCl-3) and demonstrated 22 and 11% improvement, respectively. The ion-modified electron transport layer (ETL) was deposited in the air, which reduced the power consumption of preparing perovskite solar cells (PSCs). Finally, all Q-2D PSCs were stored in the air, and three PSCs (DMF/DMSO, CsF-3, and CsCl-3) using HIS exhibited long-term stability for 1 month maintaining 80–88% of PCE, demonstrating the importance of the HIS system to improve the first step of growth orientation, which enhances the stability and photovoltaic properties of PSCs.

## Introduction

Quasi-two-dimensional (Q-2D) Ruddlesden–Popper layered metal halide perovskites are of interest because of their better photoelectric properties, higher moisture stability, and lower light-induced degradation, and they are often compared with three-dimensional (3D) perovskite materials.^1−5^ The hydrophobic organic spacer cations allow excellent interlocking between organic and inorganic substances.^6^ The general chemical formula of the Q-2D layered perovskite is A_2_B_*n*–1_M*_n_*X_3*n*+1_ (*n* = 1, 2, 3, 4, ...), where A is a long-chain organic cation (spacer) [butylammonium (BA^+^) or phenylethylammonium (PEA^+^)],^7−9^ B is a short-chain organic cation [methylammonium (MA^+^) and formamidinium (FA^+^)],^10^ M is a divalent metal cation (Pb^2+^ or Sn^2+^), X is a halide anion (I^–^, Cl^–^, or Br^–^), and n represents the number of inorganic [MX_6_]^4–^ octahedral layers separated between the interdigitating bilayers of intercalated bulky alkylammonium cations.^11,12^

The underutilization of sunlight in Ruddlesden–Popper phase Q-2D perovskites is because of their sandwich-like structure, where organic spacer cations parallel to the substrate limit the charge carrier from transferring between the adjacent inorganic octahedral layers.^11^ The strong difference in dielectric constants between the organic and inorganic layers induces the formation of excitons with considerable binding energy and low carrier mobility. This results in an increased rate of charge recombination before being collected, which hinders the perovskite solar cell (PSC) performance.^13^ Although economically viable processing conditions and environmental stability have popularized Q-2D PSCs, the photovoltaic properties of Q-2D perovskites must be optimized for innate shortcomings to achieve high power conversion efficiency (PCE).^14^ Preferred crystallographic orientations are obtained through solution-engineered modification of the crystallization process of Q-2D perovskite films (PFs), such as hot casting,^15,16^ additive engineering, and thermally aged precursor solution-treated film growth to facilitate charge transport.^8,17,18^ (PEA)_2_MA_4_Pb_5_I_16_ (PMPI) films with organic cations preferentially oriented perpendicular to the substrate were obtained by Gao et al. by controlling the growth of the intermediate phase in a cosolvent.^19^ Several methods for improving the crystal growth orientation are reported.^20−22^ Li et al. reduced the nucleation sites in the solution via thermal aging of the solution to trigger colloidal aggregation, which formed a dense Q-2D PF with 18.68% PCE.^23^ Yue et al. deposited Q-2D (AA)_2_MA_4_Pb_5_I_16_ via nontoxic acetic acid as a cosolvent. The strong power-supplying ability of acetic acid with the critical component led to iodide-induced favorable cluster aggregation. Consequently, it regulated the crystal growth with increased photovoltaic efficiency (18.55%).^24^ The study demonstrated a breakthrough in photovoltaic performance through solution improvement of Q-2D PSCs. However, two-step deposition is considered a controlled and reliable method to synthesize highly uniform perovskite components for large-scale applications.^25^ The main advantage of two-step solution deposition over one-step deposition is its reproducibility,^26^ which is one of the most critical metrics for commercialization.

Tai et al. reported the use of the Pb(SCN)_2_ precursor in preparing the CH_3_NH_3_PbI_3–*x*_(SCN)_*x*_ layer in ambient air. The PSCs fabricated with this method demonstrated improved stability in humid environments and achieved a PCE of over 15%.^27^ Lai et al. reported that Q-2D PF made with fluorinated benzylammonium iodide achieved a PCE of about 20% at a relative humidity (RH) of ∼20%.^4^ Ke et al. reported applying a layered (PEA)_2_FA_2_Pb*_n_*I_3*n*+1_ perovskite light absorber via a two-step process with enhanced stability in humid conditions for solar cell applications with 11.46% PCE. It retained 86% of the initial PCE after exposure to RH of up to 60% for nearly 900 h.^28^ Lu et al. demonstrated the growth of FA-based Ruddlesden–Popper-type perovskites M_2_FA_*n*–1_Pb*_n_*I_3*n*+1_ via a two-step sequential deposition process. FA_2_FA_*n*–1_PbnI_3*n*+1_ was formed with the benzylamine (PMAI) ratio increase in the second step with >19% PCE.^29^ Wu et al. discussed the importance of the priming layer for 3D perovskites.^30^ PbI_2_ was treated with a strong ligand solvent [dimethyl sulfoxide (DMSO)] (first step) to generate homogeneous thin films effectively. The first step of preparing primer layers using mixed immersion solvents with different solvent ratios was characterized to adjust the coordination and coverage rates of PbI_2_ and phenethylammonium iodide (PEAI).

Recently, the net-zero issue has received extensive attention. According to the Paris Agreement^31^ and the Carbon Border Adjustment Mechanism (CBAM),^32^ heavy industries, buildings, and mining should adopt comprehensive approaches to reduce emissions, and the research and industrial sectors should comply with carbon (or carbon dioxide) management in the future.^33^ The solar, renewable energy industry can control carbon emissions by replacing high-carbon emitting equipment. However, most PSCs are manufactured and stored in energy-consuming gloveboxes, which is challenging. Herein, we use nitrogen gloveboxes to control RH (25–75%) in the absence of electricity instead of the moisture-, volume-, concentration-, and humidity-controlled power-consuming equipment. We propose a two-step method for fabricating Q-2D PMPI (MA = methylammonium) as light-absorbing materials for solar cells. Perovskite and the hole transport layer (HTL) are manufactured in high RH (25 ± 5%), and the electron transport layer (ETL) is manufactured in the air. The difference in coordination between *N*,*N*-dimethylformamide (DMF), DMSO, and Pb^2+^ in the precursor was exploited in the first step to investigate the optimal mixing ratio of the HIS to obtain a high coverage and large grain size of active materials. The first step formed a vertically aligned (111) as a primer layer. MA^+^ can easily enter the primer layer and self-assemble to form the (111) oriented Q-2D (PEA)_2_MA_4_Pb_5_I_16_ film in the second step owing to the layered structure. We have previously reported the post-treatment of perovskite with ionic solutions to enhance electrical properties.^34^ In this study, SnO_2_ as the ETL was optimized by using ultralow-temperature ionic solutions (CsF and CsCl) to improve the carrier concentration of SnO_2_. Q-2D (PEA)_2_MA_4_Pb_5_I_16_ (PMPI) films with few defects, large grains, and a regular arrangement were obtained in the HIS. The photovoltaic efficiency (13.09%) of the solar cell was highly improved, with a 57% increase in the PCE compared to that of the DMSO-based PSC (8.3%). The PCEs of CsF- and CsCl-modified ETL-based PSCs were 16.02 and 14.58%, respectively. The durability of the device with the optimized structure was 4 weeks and maintained a higher PCE of 81–87% than those of PSCs based on a single solvent.

## Experimental Section

### Materials

The chemicals were used as purchased. Tin oxide (SnO_2_, 15% in H_2_O colloid dispersion) was purchased from Alfa Aesar. Cesium fluoride (CsF, 99%), cesium chloride (CsCl, 99%), cesium bromide (CsBr, 99%), and cesium iodide (CsI, 99%) were purchased from Sigma-Aldrich. Methylammonium iodide (MAI, >98.0%) was purchased from TCI. Lead iodide (PbI_2_, 99.9985%) was purchased from Alfa Aesar. Phenethylammonium iodide (PEAI) was purchased from Greatcell Solar. Spiro-OMeTAD was purchased from Sigma-Aldrich. 4-*Tert*-butylpyridine (TBP, >96%) was purchased from TCI. The patterned ITO glass (∼15 Ω sq^–1^) was obtained from Lumtec Taiwan, and the ITO substrate was cut into sizes of 2 cm × 3 cm for the experiment.

### Solution Preparation

The precursor solution was prepared by diluting 0.5 mL of SnO_2_ colloidal solution (15%) in 2 mL of deionized water. 7.5 mg of CsCl, CsBr, CsI, and CsF were dissolved in the dilute SnO_2_ solution to obtain CsF, CsCl, CsBr, and CsI (wt %) of 3 mg mL^–1^, respectively. The Spiro-OMeTAD solution was prepared by mixing 1 mL of Spiro-OMeTAD solution (72.3 mg of Spiro-OMeTAD powder in 1 mL of chlorobenzene) and 17.5 μL of Li-bis(trifluoromethylsulfonyl)imide (TFSI) solution (520-mg of Li-TFSI powder in 1 mL of acetonitrile). 28.75 μL of tributyl phosphate (TBP) was added to the solution.

### Fabrication of the PSC

**Figure 1:**
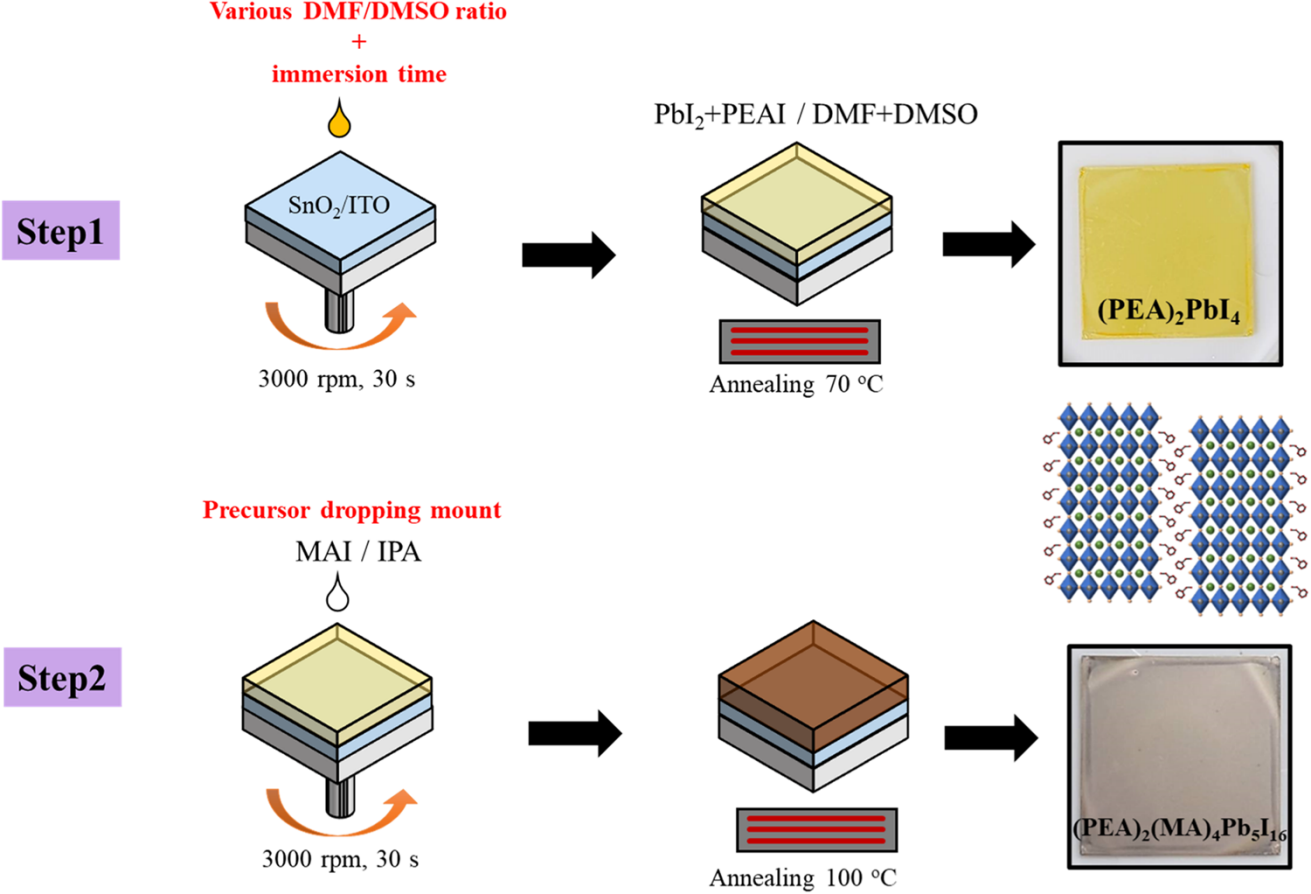
Schematic illustration of the two-step fabrication process for the Q-2D PMPI perovskite under ambient air conditions with high RH.

The glass substrate was ultrasonically cleaned sequentially by using acetone, ethyl alcohol, and deionized water for 15 min. The substrate was dried using N_2_ gas, and the glass was treated with O_2_ plasma for 3 min to remove residual contaminants adhering to the ITO glass surface. The improved surface wettability of the substrate achieved a better solution coverage for the spin-coating procedure. The SnO_2_ ETL was fabricated using the solution method. The precursor was prepared by diluting SnO_2_ solution (15%) in deionized water and adding CsF and CsCl to achieve different weight percentages of interest. The concentrations of CsF and CsCl used in this experiment were 1, 2, 3, 4, and 5 mg mL^–1^. After that, the solution was spin-coated on precleaned ITO glass, followed by annealing in air at 60 °C for 30 min to obtain a modified ETL. The ETL surface undergoes 3 min of O_2_ plasma treatment again. The absorbing layer in this study, Q-2D (PEA)_2_MA_4_Pb_5_I_16_ PF, was deposited on the ETL via the two-step solution method. In the first step, the precursor solution was prepared by dissolving PbI_2_ and PEAI with mixed solvents of various volume ratios of HIS at a stoichiometric ratio of 5:2 (i.e., 1.0 M Pb^2+^ concentration). The optimal ratio of DMF/DMSO was 8:2 in this experiment. The precursor solution was spin-coated on an ETL glass at 3000 rpm for 30 s. The deposited films were annealed on a hot plate at 70 °C for 10 min and cooled to room temperature at 25 °C. The yellow intermediate films comprising a mixture of (PEA)_2_PbI_4_ and PbI_2_ were obtained after the first-step process, as shown in Figure 1. In the second step, MAI was dissolved in an isopropyl alcohol solution (20 mg mL^–1^). The substrate was spun at 3000 rpm for 30 s. At the 15th second, a certain amount of the MAI solution (80–170 μL) was dripped on the substrate slowly. Finally, the PFs were annealed at 100 °C for 20 min. The color of the obtained films turned from yellow to brown, indicating the formation of Q-2D (PEA)_2_MA_4_Pb_5_I_16_ PF. The two-step process flow is shown in Figure 1. Spiro-OMeTAD was used as the HTL to fabricate the Q-2D PSCs. The precursor solution was spin-coated on the Q-2D perovskite layer at 4000 rpm for 30 s to obtain the HTL layer. A 100-nm-thick Au electrode was thermally coated on the HTL layer.

### Measurement and Characterization

The SnO_2_-based ETL and Q-to-two-dimensional PFs morphology were analyzed via SEM to investigate the influence of different solvent ratios in precursor solutions. EDS was used to analyze the elements in Q-2D PFs to confirm the formation of Q-2D (PEA)_2_MA_*n*–1_Pb*_n_*I_3*n*+1_ perovskites. A contact angle was formed when water was dipped on a solid surface, which quantified the wettability of the SnO_2_-based ETL surface. The contact angle becomes smaller when water spreads across the surface, indicating a hydrophilic surface. By contrast, the contact angle > 90° indicates a hydrophobic surface. GIXRD was conducted to observe the crystalline structure of the Q-2D PFs with a glancing angle of 1°. The X-ray source was Cu Kα, and the scanning speed was 0.1° s^–1^ with a step size of 0.015°. The full width at half-maximum (FWHM) was analyzed using ORIGIN Gaussian fitting. UV–vis was used to measure the transmittance of the SnO_2_-based ETL and the absorbance of the Q-2D PFs. The transmittance of the SnO_2_-based ETL played a crucial role in determining the total amount of light absorbed by the absorbing layer. Steady-PL measurement was conducted by using the source wavelength (nm). PL intensity exhibits electron transfer efficiency from the PL to the ETL. AFM was used to observe the surface roughness of the PFs by scanning a tiny cantilever over the sample and measuring the forces between the probe and the surface. XPS was conducted for element composition analysis in the PFs by measuring the kinetic energy of the photoemitted electrons from the surface after the energy of incident X-rays. The Hall measurement system consists of an electromagnet, a Source Measurement Unit (SMU) instrument (KEITHLEY Model 2400), and a four-probe holder. The *I*–*V* characteristics of the Q-2D PSCs were measured under air mass (AM) 1.5G irradiation (100 mW cm^–2^) at room temperature. The PCE and FF of the solar cells were derived from the result. The external quantum efficiency (Optosolar) was measured using the SRF50 system. TRPL was conducted by using a microphotoluminescence system (Spectra Physics).

## Results and Discussion

### Vertical Q-2D (PEA)_2_MA_4_Pb_5_I_16_ (PMPI) Perovskite Morphology of the First Priming Layer in 20–30% RH

**Figure 2:**
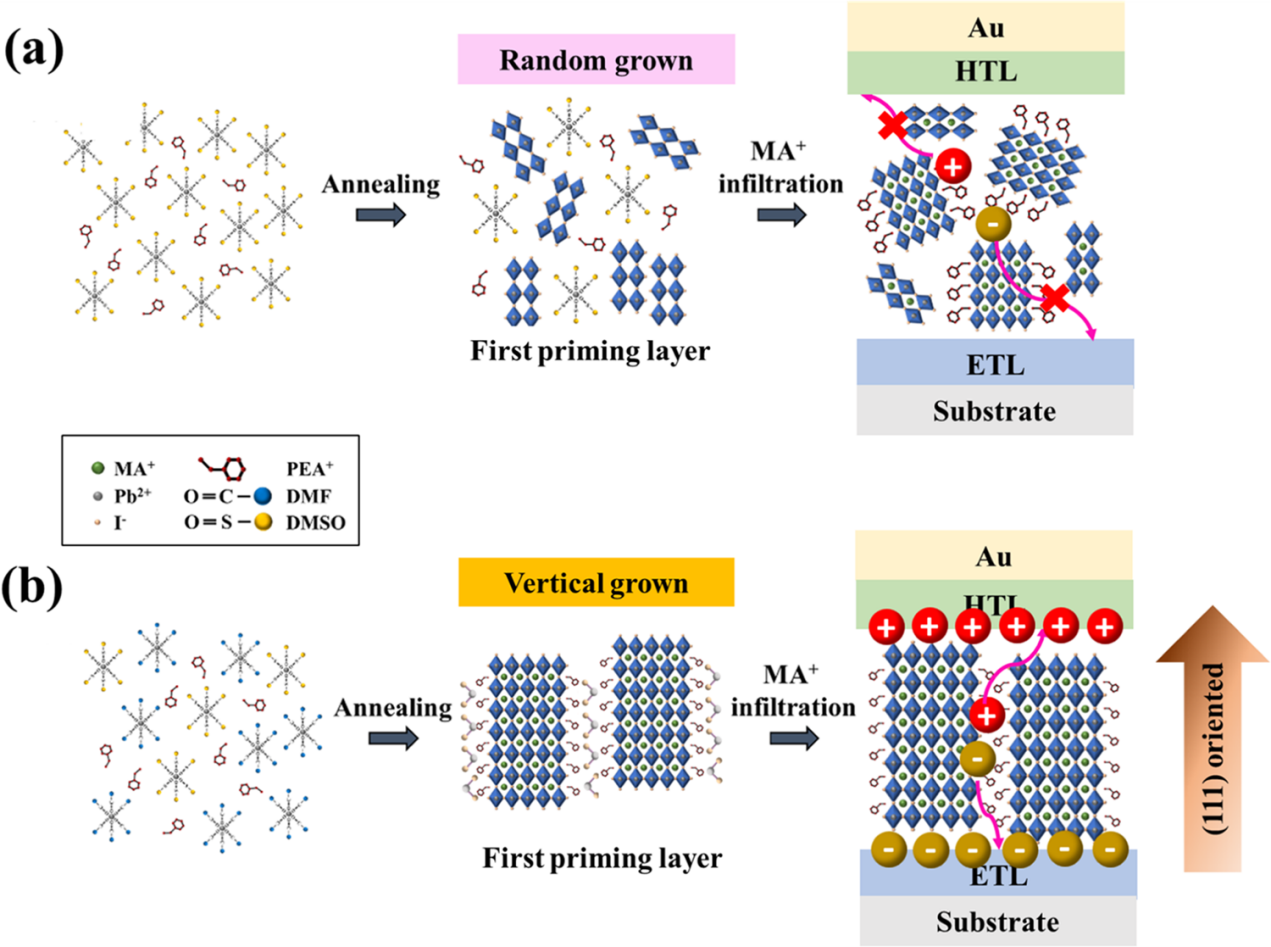
Schematic diagram of the preferential vertically oriented crystallization mechanism and charge-transport channels based on the first priming layer and Q-2D (PEA)_2_MA_4_Pb_5_I_16_ films deposited using (a) DMSO and (b) HIS (DMF/DMSO = 8:2) system.

According to previous reports, high-performance PSCs can be achieved by fine-tuning different ratios of the HIS to control the perovskite crystal growth direction and the micron-sized grain growth.^24,35^ The two-step synthesis method creates a reliable morphology of the first primer layer, directly affecting the final performance of the PSCs. We investigated different ratios of DMF to DMSO to form a high-quality first priming layer during the first step in a high RH. The reaction kinetics of the Q-2D PFs are related to the interaction between PbI_2_ and the coordinating agent (DMF or DMSO). Although single solvents can react with PbI_2_ and PEAI to form the first primer layer, the 3D perovskite structural system shows that DMSO has a high affinity for metal halides.^30,36^ The coordination of DMSO to Pb^2+^ in the precursor is strong, which might retard the reaction between PbI_2_ and PEAI with excess time to release Pb^2+^ and form the first priming layer.^37^ The slowed crystallization process makes it difficult for Pb^2+^ to react with other precursors, such as PEA^+^, affecting the coverage and forming irregular perovskite structures comprising unwanted PbI_2_.^19^ The structural mismatch in the Q-2D perovskite layer also restricts charge transfer, which is detrimental to the photovoltaics of PSCs (Figure 2a). Alternately, DMF interacts weakly with Pb^2+^ in the precursor solution, accelerating the reaction between Pb^2+^ and PEA^+^ to produce perovskite grains with small sizes and amorphous structures.^38^ Adjusting the coordination of DMF/DMSO can selectively induce the crystal growth of the intermediate phase of PFs with a highly preferred (111) orientation usually perpendicular to the substrate surface (Figure 2b), can increase crystallinity and grain sizes, reduce dielectric confinement effects due to the introduction of large organic cations, and enhance charge transfer.

### Two-Step Controlled Deposition of the Active Q-2D PMPI Absorption Layer

The uniform covering of the active layer is crucial for the design of highly efficient PSCs. An extended evaporation time localizes solute accumulation with the incomplete coverage of the perovskite layer.^39^ Therefore, the effect of the precursor solvent composition on the growth of the Q-2D PMPI crystals was explored. The first priming layer was prepared in a HIS to obtain the best deposition quality and high coverage. The dissolution of the precursor solution in pure DMF is faster than that in pure DMSO owing to the higher polarity of DMSO than DMF (relative solvent polarities of DMSO and DMF are 0.444 and 0.386, respectively).^19^ The strong coordination between excess DMSO and Pb^2+^ initiates the first step of dissolving the PbI_2_/PEAI precursor, as shown in Figure S1a. The first priming layer comprising pure DMF immediately turned yellow during annealing at 70 °C compared to the first priming layer comprising pure DMSO, which further verifies that the strong polarity of DMSO retards the crystallization rate (Figure S1b). Q-2D PFs were deposited under general acrylic glovebox (RH = 25 ± 5%) conditions rather than under energy-consuming pressure vacuum glovebox (RH < 1 ppm) conditions to investigate the effect of the HIS on PFs and reduce carbon emissions. The first priming layer was characterized to study the influence of the HIS on the formation of grain structures. Figure S2a,b demonstrates that the first priming layer was obtained with DMF:DMSO = 10:0 and 8:2, and immersion time ranging from 5 to 25 s has an optimal 100% coverage of the indium titanium oxide (ITO)/glass substrate (ImageJ Software Analysis). However, increased DMSO content makes the film cover unstable and sensitive to immersion time. The homogeneous substrate coverage of the films obtained by immersing in pure DMF is attributed to the high volatility of DMF (boiling point = 153 °C) compared to DMSO (boiling point = 189 °C).^40,41^

**Figure 3:**
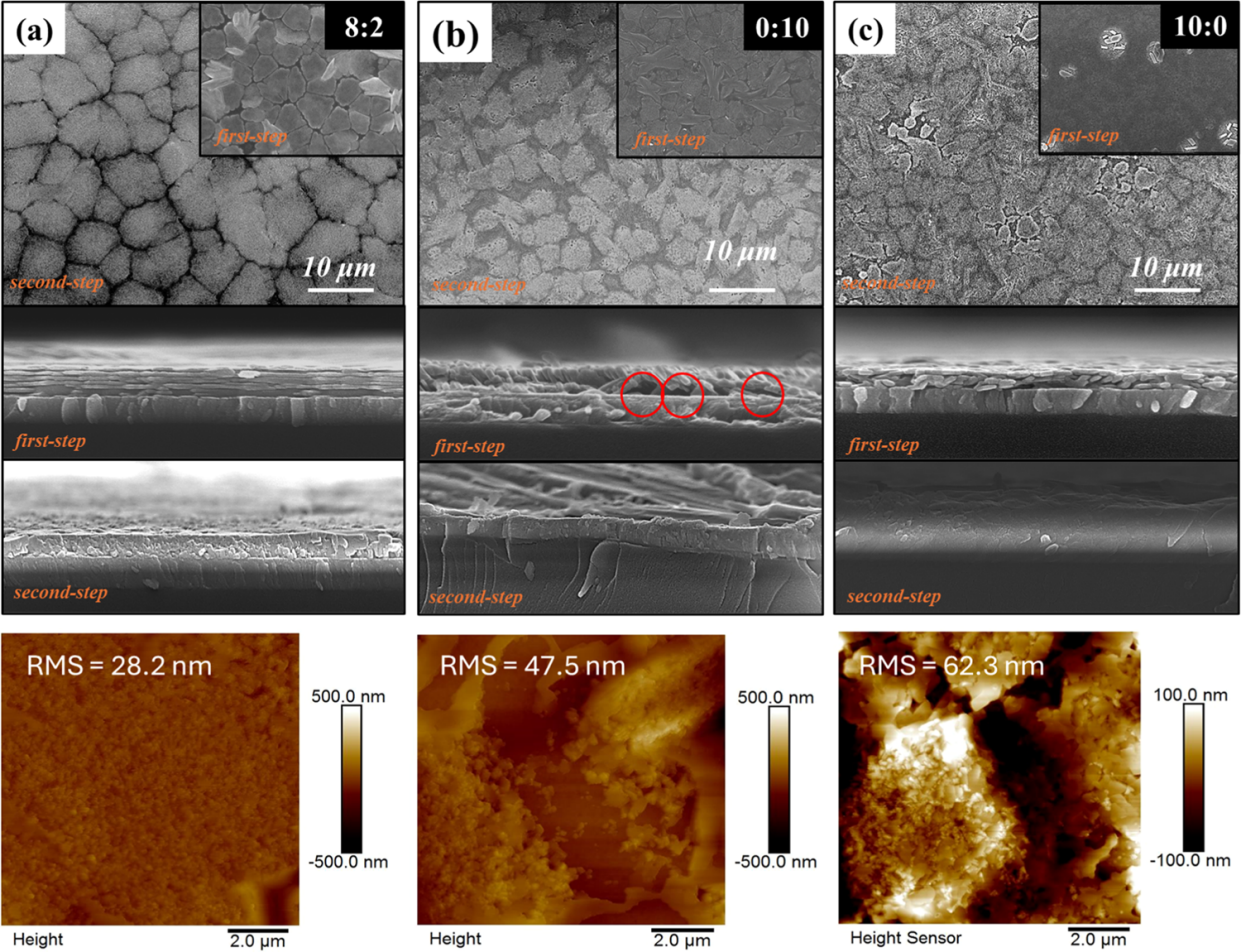
Top-view and cross-sectional SEM and AFM images of the first priming layer. Q-2D (PEA)_2_MA_4_Pb_5_I_16_ films were obtained using (a) DMF/DMSO = 8:2, (b) pure DMSO, and (c) pure DMF.

Figure 3 presents the top-view and cross-sectional images of the sample analyzed via scanning electron microscopy (SEM), and the inset shows the first step of preparing the priming layer. The inset in Figure 3a–c shows a standard Q-to-2D film laminate structure with an 8:2 (DMF: DMSO) ratio of the HIS to form high-quality films with a dense and uniform surface morphology. By contrast, cracks with a visible number of pinholes (red circles) and irregular structures are observed in the pure DMSO-based film, while the pure DMF-based films show no discernible structure, which is consistent with a high disorder. The HIS strongly coordinates to form stable intermediate films because it can retard the reaction rate between PEA and PbI_2_ in the intermediate film. The first priming layer prepared in DMF:DMSO = 8:2 has a nanoporous morphology as observed from magnified images (Figure S3a), which is favorable for MA^+^ penetration and embedding in the second step of the process.^28^ Therefore, HIS instead of a single solvent is necessary to obtain well-crystallized Q-2D PF while maintaining a good substrate coverage in the subsequent process. The ultraviolet–visible (UV–vis) absorption spectrum (Figure S3b) of the Q-2D PF shows high-energy continuous absorption edges and low-energy exciton peaks corresponding to its lowest exciton resonance. Its absorption peak near 520 nm corresponds to the bandgap excitation properties of (PEA)_2_PbI_4_.^42−44^ The crystallization of the first priming PFs prepared in the HIS with different solvent ratios was investigated via grazing incidence X-ray diffraction (GIXRD) pattern analyses (Figure S3c). The first primer layer shows distinct peaks at diffraction angles 2θ = 10.8, 16.2, 21.8, 27.33, and 36.2° indexed as a series of reflections of (004), (006), (008), (0010), and (0012) of (PEA)_2_PbI_4_, respectively. The results indicate the formation of layered Q-2D PF in the first step, similar to the previously reported XRD patterns of Ruddlesden–Popper crystals with PEAI as the sizable organic cation.^28,45−47^

Figure 3a–c displays the top-view and cross sectional SEM and atomic-force microscopy (AFM) images of Q-2D PMPI films with different solvent-based first priming layers. The surface morphology of the DMF/DMSO-based PF has a larger structure and higher grain size (8.39 μm) than that of the DMSO-based PF (Figure S4a,b) with a more uniform cross section, consistent with fewer internal boundaries and grains with oriented stripes perpendicular to the boundaries. The DMF-based film is irregular after MAI is embedded, owing to the fast crystallization in the first step. Thus, introducing an appropriate amount of DMSO in the first step is crucial for obtaining highly oriented Q-2D crystals. However, an excessive DMSO adversely affects the growth of favorably oriented crystals. SEM images are consistent with the results of AFM measurements. The HIS-based PFs exhibit smoother morphology with lower root-mean-square (RMS) surface roughness (28.2 nm) compared to the PFs synthesized by immersing in pure DMSO and DMF with RMS values of 47.5 and 62.3 nm, respectively.

**Figure 4:**
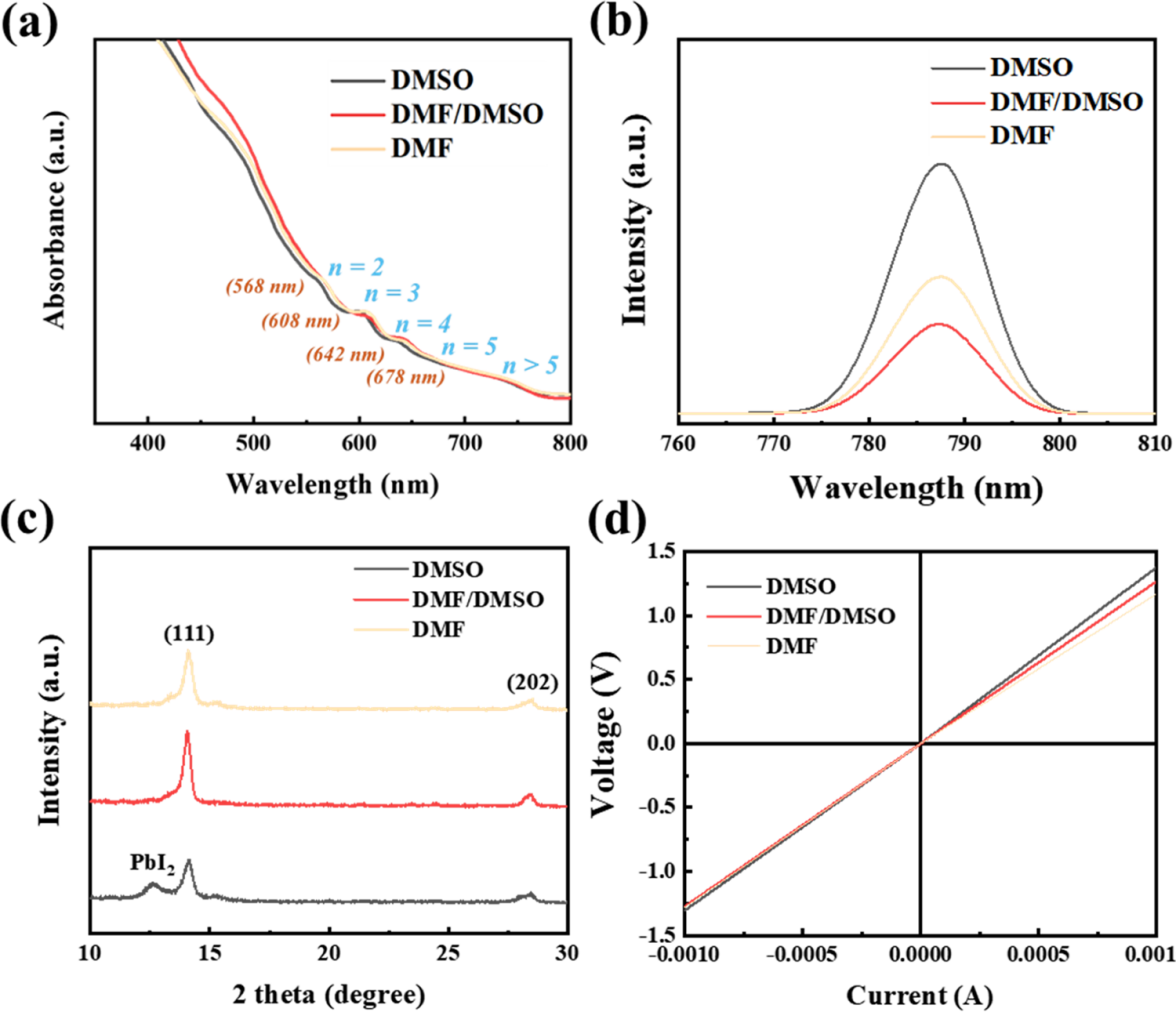
(a) UV–vis absorption spectra, (b) PL spectra, (c) GIXRD patterns, and (d) Hall measurement of Q-2D (PEA)_2_MA_4_Pb_5_I_16_ films deposited on the ITO/substrate using HISs with different solvent ratios.

Figure 4a shows the UV–vis absorption spectra of Q-2D PMPI films in HIS with different solvent ratios for investigating their internal phase distribution. The 568, 608, 642, and 678 nm absorption peaks indicate various exciton peaks associated with different high and low n-values. Thus, the film is a mixture of several *n*-values that self-organize into a quantum-well structure. The low broad absorption bands in the long wavelength region are attributed to the large *n* > 5 phase.^9,14^ The optical bandgap is obtained directly from the Tauc plot (Figure S4c). Q-2D PFs exhibit a similar optical bandgap of 1.8 eV. Figure 4b shows the photoluminescence (PL) spectra of the Q-2D PF, measured on the Q-2D PF/untreated-SnO_2_/ITO substrate. The central peak of Q-2D PMPI is located at 786 nm and is generated from the lowest bandgap emission of the high n phase. The PL intensity of the DMF/DMSO-based PF is reduced owing to the vertically aligned crystals facilitating charge transfer and collection, inducing a strong nonradiative loss of photogenerated charge carriers in the PF, which is consistent with the crystal growth mechanism shown in Figure 2b. Figure 4c indicates that the GIXRD patterns of Q-2D PMPI have strong diffraction peaks at 14.1 and 28.2° corresponding to the (111) and (202) planes, respectively. The preferred vertically aligned (111) Q-2D PMPI crystal plane has good photovoltaic properties.^8,11,15^ The first priming layer prepared in HIS has a strong intensity. The FWHM of the peak (2θ = 14.1°) in Table 1 decreases from 0.486° (DMF) and 0.437° (DMSO) to 0.326° (DMF/DMSO), indicating the formation of grain structures, consistent with the morphology changes observed in SEM images. The material is highly disordered, owing to the short crystallization process of the pure DMF-based film deposited in the first step. A relatively broad peak with a high FWHM is observed for the film embedded in the MAI solution, suggesting that Q-2D PMPI comprises unorganized oriented crystals. However, a further increase in the amount of DMSO disrupted the grain structure. The DMSO-based PF shows a distinct PbI_2_ peak at 12.7°, confirming that retarding crystallization causes incomplete film transformation. Q-2D PF crystallization depends on the first priming layer deposited in the first step. The Q-2D PMPI was successfully synthesized using a two-step method in a HIS to control the perpendicular growth direction. Improving the film quality and crystallinity improves the electronic and optoelectronic conversion efficiency, and small-angle 2D characteristic peaks are not observed in the GIXRD patterns (in Figure 4c).

**Table 1:** FWHM Extracted from Figure 4c for the Comparison of Q-2D PFs in the First Step

HIS ratio (DMF/DMSO)	FWHM (2θ = 14.1°)	FWHM (2θ = 28.4°)
0:10	0.486	0.737
8:2	0.326	0.460
10:0	0.437	0.675

Figure S5 reveals the Q-2D PF obtained after the second step with different MAI contents. Q-2D PMPI obtained by the dropwise addition of 110 μL of MAI has the best film quality with nonradiative loss, vertical orientation, and the least amount of unconverted PbI_2_. The SEM images with different magnifications further emphasize the formation of lamellar structures as a typical morphology of Q-2D layered materials (Figure S5a–d). The films exhibit square grains similar to Q-2D crystals of graphite or MoS_2_, suggesting a perpendicular growth of crystals and confirming that the Q-2D perovskite comprises stacked Q-2D solids.^48^ An increase in the MAI solution from 80 to 110 μL decreases the FWHM of the (111) peak considerably from 0.410 to 0.326 (Figure S5e and Table S1). The preferred crystal orientation on the (111) surface corresponds to the growth of Q-2D perovskite crystals perpendicular to the substrate surface, which improves the crystallinity and PCE. Alternately, an increase in the MAI solution from 110 to 170 μL increases the FWHM of (111) to 0.383, which indicates disorder in the PF. The grain boundary is undefined, indicating that the crystallinity of the obtained PF decreases. The XRD results are consistent with the PL spectra (Figure S5f). The PL intensities of the MAI drop amounts were measured on a Q-2D PF/untreated-SnO_2_/ITO substrate structure.

Figure 3 shows that the first step of depositing the first priming layer influences the final Q-2D PMPI morphology, and the HIS induces a well-crystallized orientation and film quality. We investigated a second process (MAI solution embedding) in an excessively high humid (from 30 to 60 ± 5% RH) environment to observe the effect on the successive processes of the optimized structure. The surface morphology and grain boundaries of Q-to-Tie-based PMPI deposited in highly humid conditions are distinct (Figure S6) with untransformed PbI_2_ and blurred grain-boundary structures. Preliminary demonstration of films with HIS-optimized structures fabricated at high relative humidity.

The Hall measurement was measured to investigate the electrical properties (Figure 4d and Table 2). The electron mobility values of DMSO- and DMF/DMSO-based PFs are 22.3 and 39.8 cm^2^ V^–1^ s^–1^, respectively, indicating that the HIS-based PF has the highest electron mobility, in agreement with the PL results. Adjusting the solvent ratio in the HIS can improve the charge transfer efficiency of Q-2D PFs, indicating that the grain structure of Q-2D perovskite facilitates charge carrier transport in PSCs and deposition under highly humid conditions. The grain structure can be achieved by adjusting the coordination strength between PbI_2_, DMF, and DMSO, illustrating the importance of optimizing the first priming layer. The distribution of chemical compositions throughout Q-2D HIS-based PF was analyzed via X-ray photoelectron spectroscopy (XPS). The XPS results of Q-2D PMPI show two peaks: Pb 4f_5/2_ at 143.5 eV and Pb 4f_7/2_ at 138.6 eV. The peaks at 631.2 and 619.5 eV are attributed to I 3d states and agree with the 3d_3/2_ and 3d_5/2_ states of iodide (Figure S7a,b). Furthermore, no peaks of metallic Pb are observed in the spectra, suggesting the complete conversion of PbI_2_ into perovskite.^49^ The spectra of C 1s show two peaks at 286.7 and 285.3 eV (Figure S7c). The peak with high binding energy is ascribed to C–N bonding in MA^+^, and the peak with low binding energy is from adventitious carbon.^50,51^ The results conform with the XPS result of PEA-based perovskite in other reports, confirming the formation of Q-2D PMPI.^52^

**Table 2:** Carrier Density (*n*), Mobility (μ), Resistivity (ρ), and Conductivity (σ) for Q-2D (PEA)_2_MA_4_Pb_5_I_16_ Films

samples	*n* (cm^–3^)	μ (cm^2^ V^–1^ s^–1^)	ρ (Ω cm)	σ (S cm^–1^)
DMSO	5.88 × 10^20^	22.3	4.77 × 10^–4^	2.10 × 10^3^
DMF/DMSO	4.80 × 10^20^	39.8	2.97 × 10^–4^	3.37 × 10^3^
DMF	5.94 × 10^20^	35.4	3.27 × 10^–4^	3.06 × 10^3^

### Characterization of the Carrier Recombination, Carrier Concentration, Hydrophilicity, and Grain Size of the SnO_2_-Based ETL

The ETL–PF interface should be compact and pinhole-free to avoid direct contact between the transparent conducting substrate and the active layer at the PSC interface. Charge recombination is minimized through a suitable energy-level arrangement of the ETL to achieve a high open-circuit voltage (*V*_oc_) and short-circuit current. We have reported^34^ the effect of passivation defects achieved by post-treating perovskite surfaces with different ionic solutions in the air to explore mechanisms for enhancing the electrical performance of the device. In this study, we systematically investigated the photovoltaic properties of a modified ETL synthesized by doping SnO_2_ colloidal solutions with different concentrations of CsF and CsCl ionic solutions. The ETL was annealed at 60 °C. The concentration of CsF immersion solvent is 1 mg mL^–1^, labeled below as CsF-1, and so on. Figure S8 compares the SEM top-view images of modified ETL obtained at various CsF and CsCl concentrations to the changes in grain sizes. Figure S8a–e demonstrates that the morphologies of pristine SnO_2_ and modified films are smooth and homogeneous. The average grain sizes of CsF-SnO_2_ and CsCl-SnO_2_ films increase with the increase in the concentration of the immersing solvent. The average grain size increases from 10.16 nm (CsF-0) to 14.14 nm (CsF-5) and 17.23 nm (CsCl-5), as calculated using ImageJ software (Figure S8f). The presence of Cs^+^, F^–^, and Cl^–^ ions might increase the grain size and decrease the grain boundaries of the SnO_2_-treated film.^53^ The modified SnO_2_ precursor effectively avoids aggregation and forms a film with a uniform coverage. The complete surface coverage of ITO by the ETL is the key to realizing high-performance PSCs in well interfacial contact and conduct with the perovskite layer. Conventional tin dioxide films provide nonwetting surfaces to perovskite precursor solutions and require plasma pretreatment to improve the surface energy of the ETL.^54^ The inset of Figure S8 shows the water contact angle measurements of SnO_2_ for surface hydrophilicity. The contact angle of the pristine SnO_2_ film is 44.6°, while it decreases for the modified SnO_2_ film to 3.7° (CsCl-5) and 8.3° (CsF-5). Differences in wettability affect the surface morphology of the perovskite spin-coating process,^55^ and the results suggest that CsF- and CsCl-modified ETLs have uniform coverage, nonporous morphology, and high wettability, favorable for the deposition of perovskite layers.

The ETL is compact and pinhole-free with a minimum thickness to minimize the series resistance of PSCs (Figure S9). The cross-sectional SEM images show the ETL thickness values for different concentration modifications. ETLs in all PSCs were spin-coated once on the ITO substrate. The thicknesses of CsF-0-, CsF-5-, and CsCl-5-modified SnO_2_ films are 13.8, 25.3, and 20.2 nm, respectively. Although CsF- and CsCl-modified SnO_2_ is thin and suitable as an ETL layer, the thickness of the film increases as the concentration of the ionic solution increases. We further measured dynamic light scattering (DLS) to investigate the particle size change and aggregation of SnO_2_ in the colloid solution after adding CsF and CsCl to understand the nucleation kinetics of the ETL. The average particle size of the corresponding SnO_2_ in CsF and CsCl is shown in Figure S10 and summarized in Table S2. The average particle size of SnO_2_ in the pristine colloidal solution is 11.4 nm and increases to 20.4 and 17.7 nm with increasing concentrations of CsF and CsCl, respectively. F^–^ ions strongly coordinate with Sn^4+^ and adsorbed ions increase individual grain sizes.^56^

SnO_2_ nanoparticles aggregate because the electrostatic potential of nanoparticles in colloidal solutions decreases with time. The surface ζ-potentials of CsF-5 and CsCl-5 are −13.17 and −25.56 mV (Figure S11), respectively, with an increasing additive concentration higher than that of pristine SnO_2_ (−5.35 mV). A high absolute value of ζ-potential indicates strong electrostatic repulsion between the particles in colloidal solutions, which increases stability.^57^ The electrostatic potentials of CsF-5 and CsCl-5 additives are −11.37 and −22.96 mV, respectively, after 7 days, and the original additive decreases to −1.84 mV. CsF and CsCl additives increase the electrostatic potential and antiaggregation similar to commercially available SnO_2_ solutions containing the KOH dispersant.^58^ The optimized CsCl-SnO_2_ and CsF-SnO_2_ clusters are well-controlled between 10 and 20 nm and the ideal size for a uniform SnO_2_-based ETL.^59^ The CsF- and CsCl-modified ETLs show considerably thinner thickness control, probably attributed to the well-controlled agglomeration in the solution. The annealing of SnO_2_ films aggregates SnO_2_ particles.^60^ Thus, the uniform doping of CsF and CsCl in a SnO_2_-based ETL was verified via energy-dispersive X-ray spectroscopy (EDS). The dispersion of the additives in SnO_2_ films after annealing was verified. The EDS results (Figure S12) show that CsF-3 and CsCl-3 have uniformly dispersed Cs^+^, F^–^, and Cl^–^ ions, demonstrating that additives can improve the agglomeration of SnO_2_ in colloidal dispersion^61^ and ions are successfully introduced into SnO_2_ films. No large grains (ITO substrate) are observed (Figure S8) as the aggregation of SnO_2_ particles is effectively prevented by CsCl and CsF during annealing,^57^ resulting in the formation of a uniform modified SnO_2_-based ETL film.

**Figure 5:**
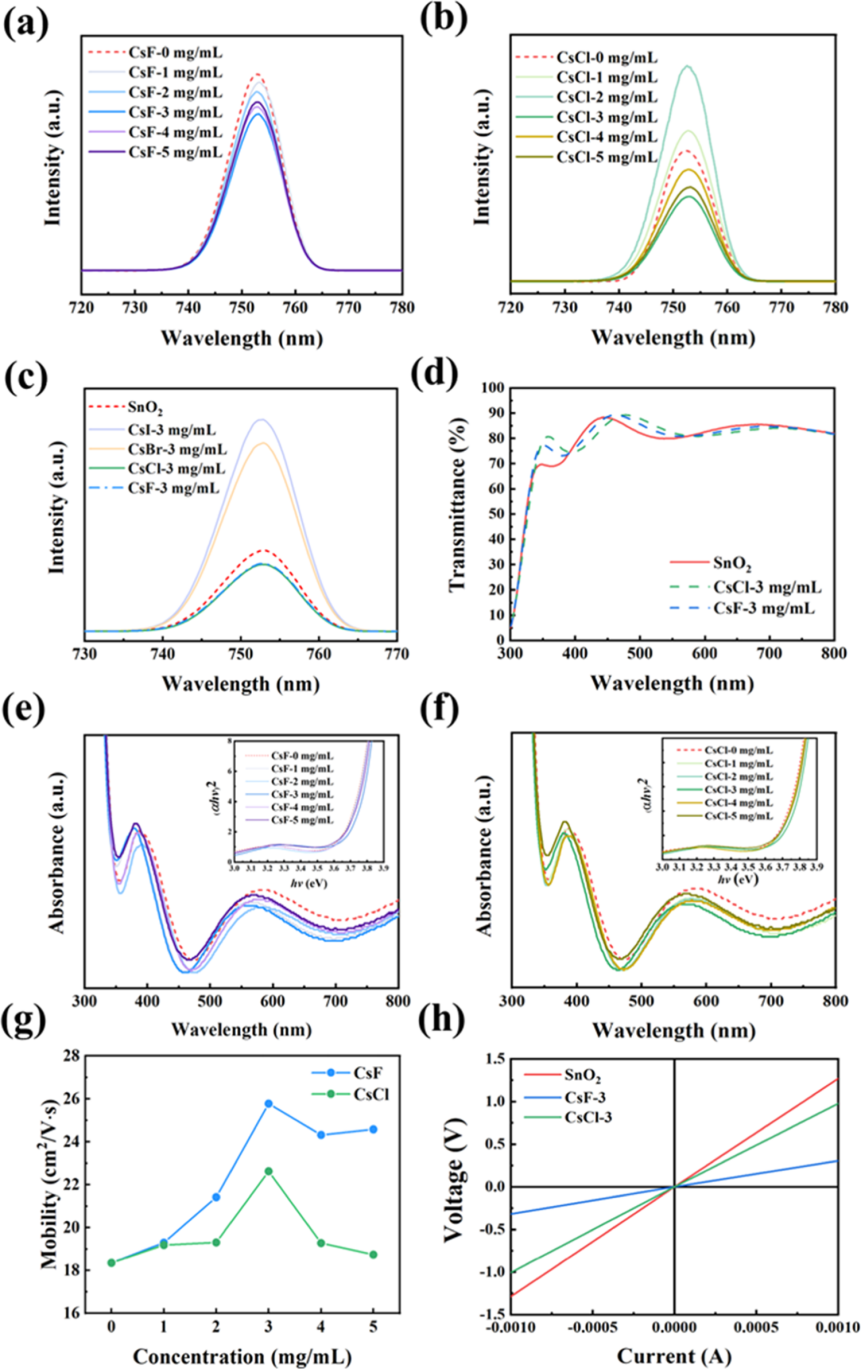
(a, b) PL spectra of the Q-2D perovskite absorber deposited on various ETLs. (c) PL spectra of SnO_2_-based ETL based on 3 mg mL^–1^ CsI, CsBr, CsCl, and CsF. (d) Transmittance spectra of the pristine and modified ETLs. UV–vis absorption spectra and corresponding Tauc plots (inset) of ETLs obtained by doping with different (e) CsF and (f) CsCl concentrations. (g) Electron mobility of SnO_2_-based ETLs with varying concentrations of CsF and CsCl. (h) *I*–*V* curves of Ag/SnO_2_/ITO, Ag/CsF-SnO_2_/ITO, and Ag/CsCl-SnO_2_/ITO structures via Hall characterization. The doping concentration is 3 mg mL^–1^.

The dynamics of the electron extraction capacity and recombination of PSCs were investigated by using steady-state PL with different concentrations of CsF and CsCl. Literature reports^53−59^ rarely compare the doping of a film with different ion solutions as most reports refer to a single ionic solution. We compared halogen-containing CsBr- and CsI-modified SnO_2_ to explore the suitable HIS system for preparing Q-2D perovskite with an appropriate ETL, as shown in Figure 5a–c. No significant shift in the PL peak position indicates that the doping ionic solution maintains consistency in SnO_2_ colloidal solutions with an optimal doping concentration of 3 mg mL^–1^. CsF-3 and CsCl-3 have the lowest PL intensity, indicating better charge transfer properties. The quality of the modified films decreases with an increasing concentration of doped F, which might be owing to the low boiling point of the complex compounds that thermally facilitate ionic diffusion during the growth of CsF-SnO_2_ and CsCl-SnO_2_ crystals.^53,62^ However, the PL intensity as shown in Figure 5c of the optimally modified CsF-SnO_2_ and CsCl-SnO_2_ ETLs is higher than that of the untreated film, which might be owing to the larger radius of Br^–^ and I^–^, making it difficult for ions to enter the SnO_2_ lattice. The transmission spectra in Figure 5d show a high transmittance of the doped films, even higher than that of the pristine SnO_2_ film in the short wavelength region beneficial for the fabrication of PSCs before and after introducing CsF and CsCl into the SnO_2_-based ETL on the ITO. The high transmittance of the ETL allows more incident light to reach the absorber layer, which improves the light-harvesting efficiency.

The charge carrier dynamics and performance of PSCs have a strong relation with the bulky electrical property of the ETL. The optical bandgap of the ETL directly determines the electron extraction efficiency from the PF. CsF-SnO_2_ and CsCl-SnO_2_ ETLs show absorption edges at ∼370 nm in the UV–vis absorption spectra (Figure 5e,f). The resulting band gaps are summarized in Table S3. The band gaps are slightly wider with an increasing concentration of CsF and CsCl up to 3 mg mL^–1^ owing to the Burstein–Moss effect, which describes the function between the up-shifted Fermi level and increased carrier concentration caused by the filling of states in the conduction band.^63^ An increase in CsF and CsCl concentrations narrows the bandgap owing to the many-body interaction effect by F^–^ and Cl^–^ with free carriers.^64^ The electron mobility depends on grain boundaries and ionized impurity migration based on the scattering mechanism.^65^Figure 5g compares the mobility of the Hall characterization of CsF-3 and CsCl-3 films with different concentrations. The electron mobility of CsF-0 is 18.35 cm^2^ V^–1^ s^–1^. The electron mobility follows a trend similar to that of the carrier concentration and Fermi-level shift as the concentration increases with the high values of 25.78 and 22.63 cm^2^ V^–1^ s^–1^ for CsF-3 and CsCl-3, respectively. Table S4 summarized the detailed carrier density (*n*), electron mobility (μ), and Fermi-level changes (Δ*E*_f_) of all modified SnO_2_ films from Hall measurement. Au/SnO_2_/ITO device structures were fabricated under various conditions to evaluate their electrical conductivity (Figure 5h). The conductivities (*I*–*V* curves) of CsF-SnO_2_ and CsCl-SnO_2_ ETLs are higher than that of the pristine SnO_2_ ETL, indicating that the current density of the modified SnO_2_ devices is higher than that of the pristine SnO_2_ devices at the same bias voltage, providing an efficient pathway for electron transfer. We compared the Q-2D PMPI morphology deposited on CsF-3-SnO_2_ and CsCl-3-SnO_2_ ETLs in Figure S13a,b. Dense and continuous Q-2D PFs are formed on both ETLs. The average crystal sizes of the calcite films deposited on CsF-3-SnO_2_ (10.32 μm) and CsCl-3-SnO_2_ (9.31 μm) are larger than that of the films on unmodified SnO_2_, which was 8.39 μm (Figure S13c,d). The micron-level grain growth benefits from the modified ETL,^66^ and the improvement of the grain boundaries of the active layer also favors the charge transport in PSCs.

### Photovoltaic Characterization and Durability of PSCs

**Figure 6:**
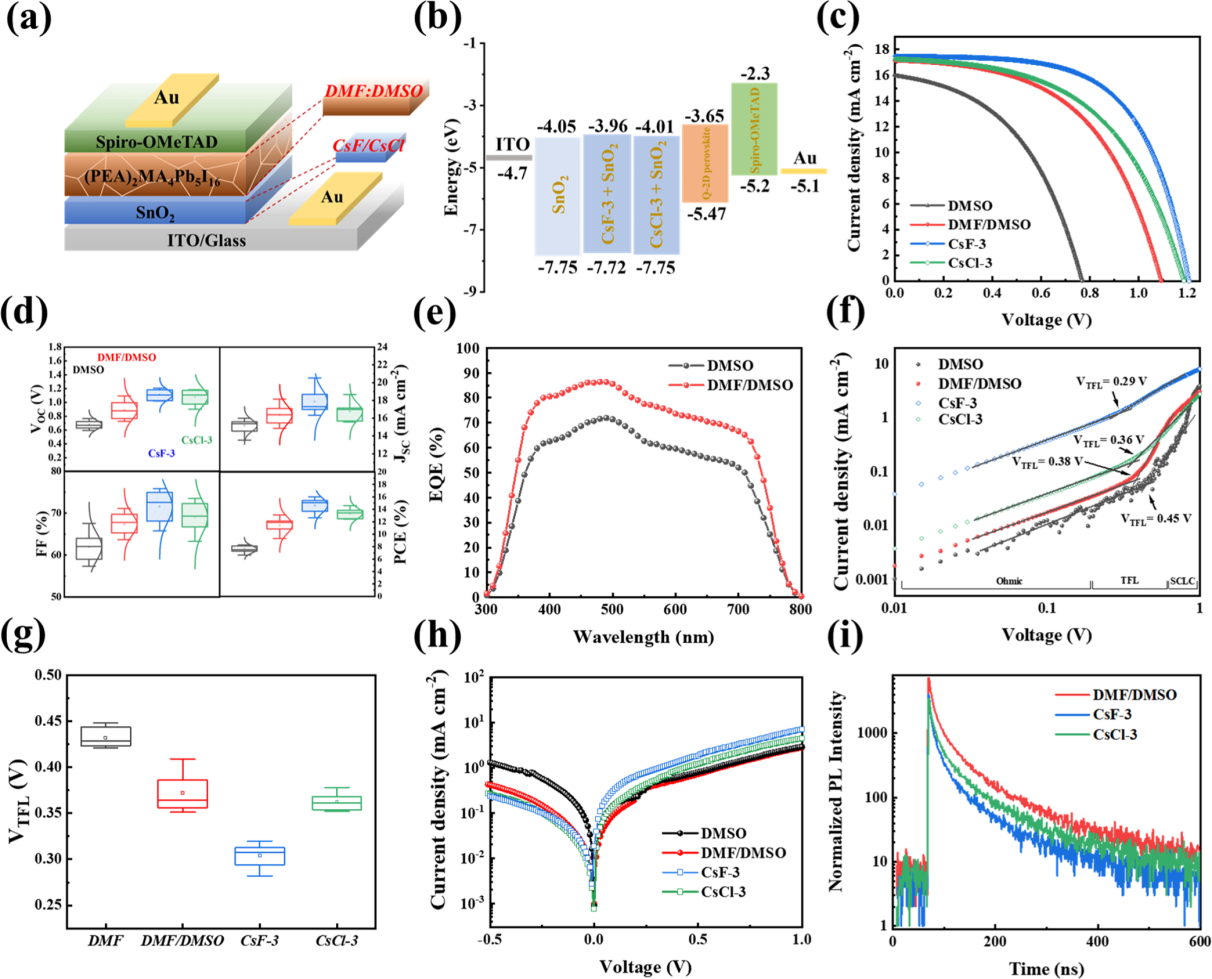
(a) Q-2D PSC structure. (b) Energy band diagram of Q-2D PSCs. (c) *J*–*V* curves for the optimized planar PSCs under an AM1.5G solar simulator using Q-2D (PEA)_2_MA_4_Pb_5_I_16_ perovskite films formed using HISs with different solvent ratios and modified ETLs. (d) Statistical distributions of *V*_oc_, *J*_sc_, FF, and PCE of Q-2D PSCs (data collected from 30 to 40 cells). (e) External quantum efficiency spectra of the devices. (f) Space-charge-limited current (SCLC) curves showing the typical three regions. (g) Statistical distribution of *V*_TFL_ from SCLC curves (data collected from 10 cells). (h) Dark *J*–*V* curves of various Q-2D PSCs. (i) TRPL spectra of Q-2D (PEA)_2_MA_4_Pb_5_I_16_ films deposited on the pristine SnO_2_, CsF-3-SnO_2_, and CsCl-3-SnO_2_ ETLs.

The planar structures of Au/spiro-MeOTAD/Q-2D PMPI/CsF-SnO_2_ or CsCl-SnO_2_/ITO substrates were considered to investigate the effects of the HIS with different solvent ratios and ETL modifications on the photovoltaic performance (Figure 6a). Based on previous reports, the corresponding flat-band energy levels are shown in Figure 6b.^6,9,28,66^ The structure of Au/spiro-MeOTAD/Q-2D PMPI (using DMSO)/SnO_2_/ITO substrates is labeled as DMSO; the structure of Au/spiro-MeOTAD/Q-2D PMPI (using DMF/DMSO= 8:2)/SnO_2_/ITO substrates is labeled as DMF/DMSO; the structure of Au/spiro-MeOTAD/Q-2D PMPI (using DMF/DMSO= 8:2)/CsF-3 mg mL^–1^-SnO_2_/ITO substrates is labeled as CsF-3; and the structure of Au/spiro-MeOTAD/Q-2D PMPI (using DMF/DMSO= 8:2)/CsCl-3 mg mL^–1^-SnO_2_/ITO substrates is labeled as CsCl-3. The champion performance photocurrent density–voltage (*J*–*V*) characteristic curves of PEA-based Q-2D PSCs were measured under standard AM1.5G illumination (100 mW cm^–2^) (Figure 6c). The corresponding photovoltaic parameters are summarized in Table 3. The PSC prepared from the DMSO-based Q-2D PMPI has the following parameter values: short-circuit current (*J*_sc_) = 15.99 mA cm^–2^, open-circuit voltage (*V*_oc_) = 0.768 V, fill factor (FF) = 0.6759, and PCE = 8.28%. The PCE of PSCs prepared in the HIS increased from 8.28 to 13.09%, an improvement of >55%. The increase in efficiency is mainly attributed to the enhancement of *V*_oc_ and *J*_sc_, which is consistent with the observed improvement in film quality and photovoltaic properties. We collected data from 30 to 40 devices for each PEA-based sample (Figure 6d) and observed a statistically significant improvement in the device performance after the addition of DMSO to the first priming layer in the HIS: the average *V*_oc_ increased from 0.689 to 0.903 V, FF from 63.7 to 67.2%, *J*_sc_ from 15.76 to 16.89 mA cm^–2^, and PCE from 7.89 to 11.82% with an average PCE improvement of 49%. The improved photovoltaic performance of PEA-based Q-2D PSCs can be attributed to the improved quality of the Q-2D PMPI film owing to the first priming layer-controlled crystal growth method, such as the increased grain size and improved crystal orientation.^8,11^

**Table 3:** Photovoltaic Performance of Cells

	*V*_oc_ (V)	*J*_sc_ (mA cm^–2^)	FF (%)	PCE (%)	*J*_sc_^a^ (mA cm^–2^)	PCE^b^ (%)	avg. PCE (%)
DMSO	0.768	15.99	67.59	8.3	15.86	8.23	8.12 ± 0.31
DMF/DMSO	1.093	17.15	69.83	13.09	17.02	12.99	12.81 ± 0.38
CsF-3	1.206	17.47	76.1	16.02	17.38	15.95	15.83 ± 0.56
CsCl-3	1.185	17.26	71.3	14.58	17.12	14.46	14.11 ± 0.52

a Calculated current density derived by integrating the EQE spectrum.

b PCE corrected by the current density obtained through the EQE spectrum.

Introducing CsF and CsCl via annealing at ultralow temperature into the SnO_2_-based ETL exhibits better photovoltaic performance than that of the unmodified SnO_2_-based PSCs. The PSCs of CsF-3 and CsCl-3 show the best performance with the highest PCE of 16.02 and 14.58%, respectively. The CsF-3-SnO_2_-based PSC has a *V*_oc_ of 1.206 V, *J*_sc_ of 17.47 mA cm^–2^, FF of 76.1%, and PCE of 16.02%, which can be attributed to the improved electron extraction efficiency. By contrast, the CsCl-3-SnO_2_-based PSC exhibits poorer performance than the CsF-3-SnO_2_-PSC owing to the poor electronic properties of CsCl-SnO_2_. This shows the relation between photocurrent and the Fermi level of the ETL. The up-shifted Fermi level with the introduction of CsF and CsCl can decrease the energy band offset between the ETL and active layer, inducing an increasing *V*_oc_.^67^ The high electron extraction efficiency of CsF-SnO_2_ and CsCl-SnO_2_ is attributed to the enlarged *J*_sc_.

External quantum efficiency was measured to study the nature of the *J*_sc_ enhancement in the DMF/DMSO-based PSC, as shown in Figure 6e. The spectral response of the DMF/DMSO-based PSC with the grain structure is higher than that of the DMSO-based PSC, suggesting that the PSC has a high photon-to-electron conversion efficiency. This improvement is related to optimizing the Q-2D PMPI crystal orientation, which efficiently converts to charge carriers and is collected by the terminal electrode. Notably, Q-2D PFs have a low external quantum efficiency in the long wavelength region. This result is consistent with the UV–vis absorption spectrum in Figure 4a.

The SCLC technique was used to investigate the changes in the electron trap density of the PSC after structural optimization and the ETL modification of Au/Q-2D packaged crystal/ETL/ITO structured devices, as shown in Figure 6f. The *J*–*V* curves change from the low-voltage linear region (ohmic region) to the trap-filling limit region and finally reach the SCLC region.^68^*N*_trap_ is evaluated by the starting voltage in the trap-filling limit (TFL) region (*V*_TFL_). Carrier mobility is derived from the SCLC region. The trap density (*N*_trap_) is calculated using eq 1(69)
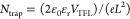
1where *N*_trap_ is the trap density, ε_r_ is the relative dielectric constant,^70^ ε_0_ is the vacuum permittivity (8.85 × 10^–12^ F m^–1^),^69^*e* and *L* are the elementary charge and thickness of the PF, respectively.^71^ The increased current in the TFL region can be attributed to the continuously filled trap states until they reach the trap-filled limit voltage (*V*_TFL_). The electron trap density of the DMF/DMSO-based PSC (*V*_TFL_ = 0.38 V) active layer is lower than the DMSO-based PSC (*V*_TFL_ = 0.45 V) with calculated electron trap densities of states of 7.42 × 10^15^ and 6.26 × 10^15^ cm^–3^, respectively. HIS-based Q-2D PFs considerably reduce the defects in the active layer by improving the lattice orientation to suppress trap-assisted nonradiative recombination. The results correspond to films with large grain sizes and low PL intensities of Q-2D perovskites (Figures 3a and 4b). The reduction in *V*_TFL_ indicates that the Q-2D perovskite trap density is reduced by the doping of CsF on SnO_2_. Introducing CsF and CsCl into the ETL significantly decreases defect density to 4.78 × 10^15^ and 5.93 × 10^15^ cm^–3^, respectively, compared to devices based on the unmodified ETL. Alternately, the CsF-based device has the lowest *V*_TFL_ (0.29 V). Figure 6g shows that the average *V*_TFL_ measurements for 10 samples are consistent with the results presented in Figure 6f, demonstrating the repeatability of the grain structure of the PSCs. The lower trap density improves the electron transfer efficiency, which enhances the PCE in PSCs. This agrees with SCLC results, indicating that doping CsF or CsCl reduces the defects of the ETL and PFs.

Analyzing the different optoelectronic properties indicates the internal characteristics of the PSC device. The dark *J*–*V* curves in Figure 6h demonstrate the *J*–*V* characteristics of a PSC measured in the dark. Optimized PSC with a lower dark current and higher photocurrent. All optimized devices show low dark *J*_sc_, indicating low bulk defects in the Q-2D perovskite layer,^72^ suppressing charge recombination and optimizing charge extraction. The optimized device produces a high *V*_oc_ owing to leakage current suppression.^73^ CsF has the highest *V*_oc_ (1.206 V), as the additives cause the passivation of perovskites and the ETL interfaces. Thus, the defect-induced carrier generation rate is reduced, resulting in the lowest leakage currents.^66^ CsF- and CsCl-modified ETLs can effectively mitigate defects in PFs and reduce the current shunt paths.

Charge recombination in PSCs was analyzed via time-resolved photoluminescence (TRPL) decay spectra to determine the recombination kinetics of Q-2D PMPI/ETL/ITO structures, as shown in Figure 6i. The fast decay (τ_1_) lifetime is attributed to the charge extraction from the ETL, while the slow decay (τ_2_) is attributed to the recombination process inside the PF.^74^ The excitation light incident on one side of the ITO substrate measures the τ_1_ and τ_2_ atoms of Q-2D PF deposited on unmodified SnO_2_ as 35.2 and 186.2 ns, respectively. CsF- and CsCl-incorporated SnO_2_ layers have shortened τ_1_ of 10.8 and 12.3 ns, respectively. The average carrier lifetime is reduced by 30%, confirming that the modification improves the extraction and transport of photogenerated electrons and reduces the defect density. The carrier lifetimes of CsF-3 and CsCl-3 are shorter than those of unmodified SnO_2_ attributed to the formation of strong Pb–Cl and Pb–F bonds on the surface and the passivation of the trap density at the interface, which facilitates the extraction of charge carriers.

**Figure 7:**
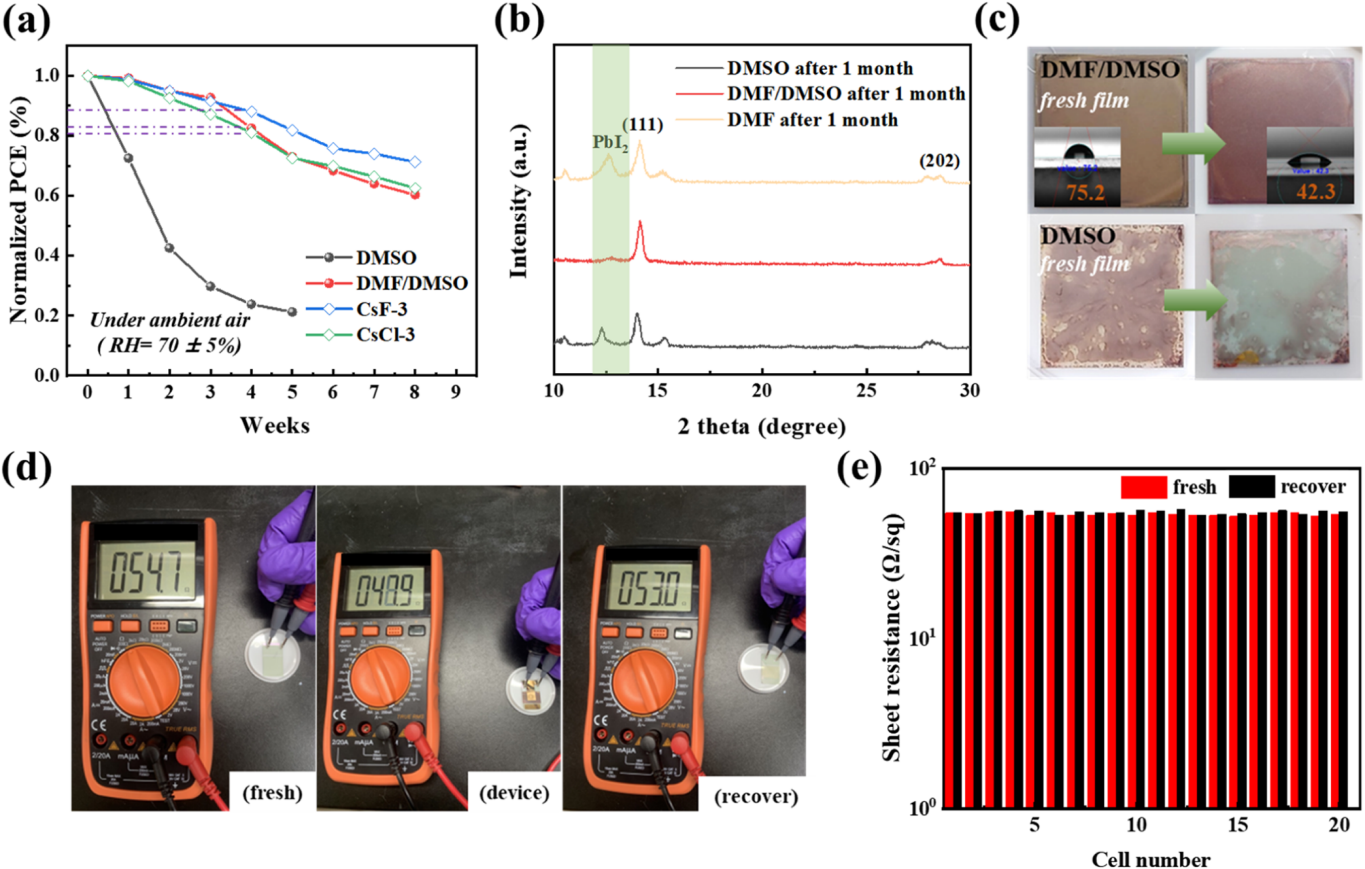
(a) Normalized PCE and long-term stability of unencapsulated Q-2D PSCs after 8 weeks of aging in the air with >65% relative humidity. (b) GIXRD patterns and (c) optical imaging of unencapsulated Q-2D (PEA)_2_MA_4_Pb_5_I_16_ films after storage in the air for 1 month with >65% relative humidity (inset shows contact angle measurements). (d) Resistance measurement of fresh and recovered ITO/glass substrates. (e) Substrate-to-substrate variation of the fresh and recovered sheet resistances for 20 different substrates.

The long-term stability of Q-2D PMPI PFs and PSC devices was investigated simultaneously. All PSCs were stored in the air with a high RH of 70 ± 5%, in darkroom and room temperature (25 °C) conditions, and aged for 8 weeks (Figure 7a). The optimized DMF/DMSO-based devices maintained ∼80–86% of their original PCE after storage in the air for 1 month (720 h). The PCE was maintained in the range 58–71% after 2 months, exhibiting excellent stability in humid conditions. Alternately, the PCE of the DMSO-based PSC device reduces to 22% after 1 min under the same storage condition. Table S5 shows earlier reports of PSCs based on various PEAI-based Q-2D perovskite materials of different systems with good PCE and long-term stability. We have improved the crystal structure of the Q-2D PMPI PF using the HIS, which hinders the reduction of the PF in moisture and allows for structural optimization before adding additives (or ionic doping) to the Q-2D absorber layer, resulting in excellent device durability. The PSC with an ion-modified ETL has excellent properties. CsF-3- and CsCl-3-based PSCs have slightly better durability than the DMF/DMSO-ETL-based PSC. Earlier reports indicate that the modification of the ETL can passivate surface defects and slow down the degradation of PCE,^54^ similar to our results. However, in this study, we consider the optimized first priming layer in the optimized structure for mitigating the degradation of the film affecting the PCE.

The GIXRD pattern and optical images of Q-2D PMPI show that DMF- and DMSO-based films stored under conditions similar to those of the PSC for 1 month (Figure 7b,c) have PbI_2_ peaks at 12.6°, indicating the rapid decomposition of unoptimized PFs. However, no noticeable material degradation is observed for the optimized DMF/DMSO-based PF with only a slight decrease in the GIXRD intensity, demonstrating its durability for storing in the atmosphere. Although Spiro-OMeTAD is a common HTL, the deterioration of HTL changes the PSC efficiency (Figure S14). The HTL was oxidized after 1 month under the same conditions, proving that the commercialization of PSCs requires the optimization of each layer.^75^ The synthesized Q-2D PSCs enable the recycling and reuse of ITO/glass substrates after cleaning with acetone or ultrasonic cleaners, reducing carbon emissions (net-zero strategy). Figure 7d,e shows a similar resistance of ITO substrates before and after cleaning of 20 cells. Thus, an HIS process approach is reported in this study that tunes the morphology of the first priming layer under high humidity while ionically modifying the ETL for passivation of defects at the bulk and layer interfaces. This affects the vertical growth and phase distribution of Q-2D PFs, which optimizes the performance of PSCs and drastically improves the durability of the PSCs in the air. This effective strategy can be applied to relevant perovskite devices such as memories, light-emitting diodes, and photodetectors. The devices are fabricated and stored in low energy-consuming devices compliant with the Paris Agreement^31^ and CBAM.^32^ The highly stable structure will promote commercialization of PSCs in the future.

## Conclusions

We have demonstrated a novel intermediate-controlled crystal growth method for synthesizing Q-2D PMPI perovskite. In the first step, the first priming layer was grown in a preferred orientation perpendicular to the substrate by appropriately adjusting the ratio of the HIS (DMF:DMSO = 8:2) with 100% substrate coverage, which greatly influenced the formation of the final Q-2D PF. The (111)-optimally oriented microstructure forms ordered, highly crystalline PFs with excellent charge transport, suppressed nonradiative recombination, and moisture-resistant capability. An appropriate adjustment of the coordination of the solvent and Q-2D compounds can effectively transform disordered grains and phase distribution into ideal grains with the micron-level grain size grown perpendicular to the substrate with relatively pure and homogeneously distributed phases. The HIS system increased the PCE of Q-2D PSCs by >55% reaching to 13.09%. Ion-modified ETLs synthesized at an ultralow temperature also increased the PCE to 16.02% without destroying the HIS system. The HIS-based first priming layer can be essential in commercializing Q-2D perovskite for highly efficient and reproducible perovskites with long-term stability (88% PCE maintained for 1 month) in other photovoltaic applications. Unpackaged PSCs, ETLs, and HTLs reduced power consumption, making the HIS Q-2D perovskite system more compatible with the future solar industry with carbon emission management.

## Supporting Information Available

FWHM of Figure S5e of Q-2D (PEA)_2_MA_4_Pb_5_I_16_ films (Table S1); ζ-potential and average colloidal diameter for fresh and aged colloidal solutions of SnO_2_ with 1, 3, or 5 mg mL^–1^ of CsF and CsCl via DLS (Table S2); band gaps (*E*_g_) of 0, 1, 2, 3, 4, or 5 mg mL^–1^ of CsF-SnO_2_ and CsCl-SnO_2_ ETLs calculated from Tauc plots (Table S3); electrical properties of pristine and different concentrations of materials doped with ion-modified ETL of 0, 1, 2, 3, 4, or 5 mg mL^–1^ of CsF-SnO_2_ and CsCl-SnO_2_ (Table S4); comparison of Au/Spiro-MeTAD/(PEA)_2_(MA)_4_Pb_5_I_16_/ETL/ITO prepared using the HIS via a two-step method with previously reported PEAI-Based Q-2D PSCs (Table S5); (a) comparison of PbI_2_/PEAI precursor dissolution speed in pure DMF and DMSO at room temperature and (b) optical images of the first priming layer crystals grown on a hot plate at 70 °C at different immersion durations in pure DMF and DMSO (Figure S1); (a) optical images of the first priming layer obtained according to different immersion times and DMF/DMSO ratios (after annealing for 10 min on a 70 °C hot plate) and (b) calculated substrate coverage of the first priming layer (first step) (Figure S2); (a) SEM image at different magnifications of the first priming layer of the nanoplates with a nanoporous surface obtained in the first step (DMF/DMSO = 8:2), (b) UV–vis spectra and (c) XRD pattern of the first priming layer obtained from the first step using HISs with different solvent ratios (Figure S3); Q-2D (PEA)_2_MA_4_Pb_5_I_16_ film grain size distribution analysis prepared using (a) HIS and (b) DMSO-based first priming layers (MAI dropping amount of 110 μL) and (c) corresponding Tauc plot of Q-2D (PEA)_2_MA_4_Pb_5_I_16_ films based on HISs with different solvent ratios (Figure S4); (a–d) top views of SEM images of the standard Q-2D structure, (e) XRD pattern and (f) PL spectra of square-shaped grains of Q-2D (PEA)_2_MA_4_Pb_5_I_16_ films with different MAI dropped amounts in the second step (Figure S5); SEM images of Q-2D (PEA)_2_MA_4_Pb_5_I_16_ fabricated in different relative humidities in the second step: (a) 30 ± 5%, (b) 40 ± 5%, (c) 50 ± 5%, and (d) 60 ± 5% RHs (Figure S6); XPS spectra of (a) I 3d, (b) C 1s, and (c) Pb 4f of Q-2D (PEA)_2_MA_4_Pb_5_I_16_ (Figure S7); SEM top-view image of SnO_2_-based ETLs with (a) CsF-0, (b) CsF-3, (c) CsF-5, (d) CsCl-3, and (e) CsCl-5 mg mL^–1^, inset shows contact angle measurement, and (f) average grain size of various ETLs (Figure S8); SEM cross-section images and thickness of SnO_2_-based ETLs modified with different ionic solutions: (a) pristine SnO_2_, (b) CsF-3, (c) CsF-5, (d) CsCl-3, and (e) CsCl-5 mg mL^–1^ (Figure S9); DLS number-average distributions and cumulative proportion of different sizes of SnO_2_ particles in (a) 0, (b) 1, (c) 3, and (d) 5 mg mL^–1^ of the CsF solution (Figure S10); ζ-potential of (a) SnO_2_, (b) CsF-5, and (c) CsCl-5 mg mL^–1^ doping concentrations (Figure S11); elemental mapping of EDS of (a) CsF-3- and (b) CsCl-3-modified ETLs (Figure S12); (a, b) SEM morphology and (c, d) grain size distribution analysis of Q-2D (PEA)_2_MA_4_Pb_5_I_16_ films deposited on CsF-SnO_2_ and CsCl-SnO_2_ ETLs (Figure S13); and optical images of HTL after aging in the air after 1 month (Figure S14) (PDF).

## Abbreviations

AFM: atomic-force microscopy
DLS: dynamic light scattering
EDS: energy-dispersive X-ray spectroscopy
ETL: electron transport layer
FF: fill factor
GIXRD: grazing incidence X-ray diffraction
HIS: hybrid immersion solvent
HTL: hole transport layer
ITO: indium titanium oxide
PCE: power conversion efficiency
PF: perovskite films
PSC: perovskite solar cell
RH: relative humidity
RMS: root-mean square
SEM: scanning electron microscopy
TFL: trap-filling limit
XPS: X-ray photoelectron spectroscopy
AM: air mass
Q-2D: quasi-two-dimensional
3D: three-dimensional
TRPL: time-resolved photoluminescence
BA^+^: butylammonium
PEA^+^: phenylethylammonium
MA^+^: methylammonium
FA^+^: formamidinium
DMSO: dimethyl sulfoxide
PEAI: phenethylammonium iodide
CBAM: carbon border adjustment mechanism
DMF: *N*,*N*-dimethylformamide
PMPI: (PEA)_2_MA_4_Pb_5_I_16_
CsF: cesium fluoride
CsCl: cesium chloride
CsBr: cesium bromide
CsI: cesium iodide
MAI: methylammonium iodide
TBP: tributyl phosphate
TFSI: Li-bis(trifluoromethylsulfonyl)imide
FWHM: full width at half-maximum
PL: photoluminescence
UV–vis: ultraviolet–visible
*J*_sc_: short-circuit current
*V*_oc_: open-circuit voltage
*N*_trap_: trap density
*V*_TFL_: trap density
ε_r_: relative dielectric constant
ε_0_: vacuum permittivity
SCLC: space-charge limited current
τ_1_: fast decay
τ_2_: slow decay
