# Supplementary material for: Intermediate-Controlled Synthesis of Quasi-2D (PEA)2MA4Pb5I16 in the 20–30% Relative Humidity Glovebox Environment for Fabricating Perovskite Solar Cells with 1 Month Durability in the Air
Source: ACS Omega. 2024 Nov 27;9(49):48374–89. doi: 10.1021/acsomega.4c06621 (PMC11635506; doi:10.1021/acsomega.4c06621)
Supplement: Supplementary file 1 — ao4c06621_si_001.pdf [file ao4c06621_si_001.pdf]

Supporting Information

Intermediate-controlled Synthesis of Quasi-2D $(PEA)_2MA_4Pb_5I_{16}$ in the 20%–30% Relative Humidity Glovebox Environment for Fabricating Perovskite Solar Cells with One Month Durability in the Air

Yen-Shuo Chen[a], *Min-Han Hsieh*[a], *Ching-Chang Lin*[b], *Yi-Cheng Huang*[a], *Shang-Yu Tsai*[a], *Fu-Hsiang Ko*[a]*

[a]Department of Materials Science and Engineering, National Yang Ming Chiao Tung University, 1001 University Road, Hsinchu 30010, Taiwan

[b]Department of General Systems Studies, Graduate School of Arts and Sciences, The University of Tokyo, 3-8-1 Komaba, Meguro-ku, Tokyo 153-8902, Japan

Corresponding author's email: fhko@nycu.edu.tw

As the volume of MAI solution dipped onto the substrate increased from 80 μL to 110 μL, the FWHM of the (111) peak in Table S1 significantly decreased from 0.410 to 0.326. Then increase the amount and raise it, indicating the disordered perovskite nanosheets.

Table S1. FWHM of **Figure S5(e)** of Q-2D $(PEA)_2MA_4Pb_5I_{16}$ Films.

MAI (μL)	FWHM (2θ = 14.1˚)	FWHM (2θ = 28.4˚)
80	0.410	0.524
110	0.326	0.460
140	0.370	0.559
170	0.383	0.549

The modified SnO_2 colloidal solutions were measured the zeta potential and average colloidal diameter in Table S2. The effect of the additive as a dispersant can be observed by fresh and 7-day aging measurements. The original SnO_2 colloidal solution, which was not modified with CsF or CsCl tended to agglomerate (after 7 days), and the average particle size increased from 11.4 to 35.3 nm.

Table S2. Zeta Potential and Average Colloidal Diameter for Fresh and Aged Colloidal Solutions of SnO_2 with 1, 3 or 5 mg mL^{-1} of CsF and CsCl via DLS.

Colloidal solution	Fresh		Aged for 7 d	
	Zeta potential (mV)	d_{AVE} (nm)	Zeta potential (mV)	d_{AVE} (nm)
SnO_2	−5.35	11.4	−1.84	35.3
CsF-1	−6.81	13.0	−5.56	14.7
CsF-3	−9.25	15.9	−8.24	18.6
CsF-5	−13.17	20.4	−11.37	22.7
CsCl-1	−9.22	12.5	−7.26	15.9
CsCl-3	−15.27	14.2	−13.28	19.8
CsCl-5	−25.56	17.7	−22.96	23.2

The blue-shift of absorption peaks can be observed after introducing the CsF and CsCl into SnO_2 films from the UV-visible spectrum (Figures 5e and 5f), indicating a slight increase for bandgaps in Table S3.

Table S3. Bandgaps (Eg) of 0, 1, 2, 3, 4 or 5 mg mL^{-1} of CsF-SnO_2 and CsCl-SnO_2 ETLs Calculated from Tauc Plots.

E_g	0	1	2	3	4	5 mg mL^{-1}
CsF	3.67	3.68	3.72	3.73	3.7	3.67
CsCl	3.67	3.68	3.69	3.71	3.69	3.69

Table S4 summarized the detailed carrier density (n), electron mobility (μ) and Fermi level changes (ΔE_f) of all modified SnO_2 films from Hall measurement. The electron mobility showed a similar trend with increasing concentration as the carrier concentration is listed below. The Hall measurement system consists of an electromagnet, a Source Measurement Unit (SMU) instrument (KEITHLEY Model 2400), and a four-probe holder. The sample was placed on a four-probe holder, and the thin film material deposited on it was brought into contact with the probes to measure its electrical properties. All CsF-SnO_2 demonstrated better mobility than CsCl-SnO_2, and CsF was more conducive to electron extraction and transport, with CsF-3 having a higher mobility of 25.78 $cm^2 V^{-1} s^{-1}$ than that of CsCl-3 (22.63 $cm^2 V^{-1} s^{-1}$). The relationship between the Hall voltage and the electrical parameters of the material can be expressed by the following equation:

$$V_H = R_H \times (\frac{I \times B}{d}) \ldots\ldots\ldots\ldots\ldots\ldots\ldots\ldots\ldots\ldots (1)$$

$$R_H = \frac{V_H \times \mathrm{t}}{I_x \times B_z} = -\frac{1}{nq} \ldots\ldots\ldots\ldots\ldots\ldots\ldots\ldots\ldots (2)$$

$$n = -\frac{1}{n \times R_H} \ldots\ldots\ldots\ldots\ldots\ldots\ldots\ldots\ldots\ldots\ldots\ldots (3)$$

$$\rho = R_s \times d \ldots\ldots\ldots\ldots\ldots\ldots\ldots\ldots\ldots\ldots\ldots\ldots (4)$$

$$\mu = -\frac{1}{n \times q \times \rho} \ldots\ldots\ldots\ldots\ldots\ldots\ldots\ldots\ldots\ldots\ldots (5)$$

where V_H was determined Hall voltage (unit: V), I represented denotes the applied current (unit: A), B was the applied magnetic field (unit: Gauss), R_H represented the Hall coefficient, q denoted the charge of the carrier, which was equal to 1.602×10^{19} coulomb (C), n was the carrier concentration of the material (unit: cm^{-3}), ρ represented the resistivity of the material (unit: Ω-cm), R_s represented the sheet resistance of the material (unit: Ω/sq.), t was the thickness of the sample. d represented the thickness of the material (cm), and μ was the carrier mobility of the material (cm^2 /V·s).

Table S4. Electrical Properties of Pristine and Different Concentrations of Materials Doped with Ion-Modified ETL of 0, 1, 2, 3, 4 or 5 mg mL^{-1} of CsF-SnO_2 and CsCl-SnO_2.

CsF–SnO_2	0	1	2	3	4	5 mg mL^{-1}
n (cm^{-3})	5.24×10^{20}	7.85×10^{20}	1.09×10^{21}	3.28×10^{21}	9.35×10^{20}	9.00×10^{20}
μ ($cm^2 V^{-1} s^{-1}$)	18.35	20.35	21.42	25.78	24.32	24.58
ΔE_f (meV)	-	6.5	12.6	23.2	8.6	7.6
**CsCl–SnO_2**	**0**	**1**	**2**	**3**	**4**	**5 mg mL^{-1}**
n (cm^{-3})	5.24×10^{20}	9.54×10^{20}	9.61×10^{20}	1.01×10^{21}	9.62×10^{20}	9.58×10^{20}
μ ($cm^2 V^{-1} s^{-1}$)	18.35	19.19	19.31	22.63	19.28	19.25
ΔE_f (meV)	-	15.5	15.7	17.0	15.7	15.6

As shown in Table S5, many studies reported the demonstration of PSC based on various Q-2D perovskite materials and some of which showed good properties in terms of PCE, Voc and long-tern PCE.

Table S5. Comparison of Au/Spiro-MeTAD/$(PEA)_2(MA)_4Pb_5I_{16}$/ETL/ITO Prepared Using the HIS via a Two-Step Method with Previously Reported PEAI-Based Q-2D PSCs.

Cell structure	Method	PCE (%)	Storage humidity	Aging time	Normalized PCE	Reference
Al/Bphen/PCBM/$(PEA)_2(MA)_3Pb_4I_{13}$/PEDOT:PSS/ITO	one-step	9.4	60%	720 h	0.7	[5]
Ag/PCB/$PC_{61}BM$/$(PEA)_2(MA)_4Pb_5I_{16}$/PEDOT:PSS/ITO	one-step	13.2	45% ± 5%	960 h	0.7	[6]
Ag/BCP/$PC_{61}BM$/$(PEA)_2(FA)_4Pb_5I_{16}$/PEDOT:PSS/ITO	one-step	12.8	30% ± 5%	500 h	0.7	[7]
Ag/PEI/$PC_{61}BM$/$(PEA)_2(MA)_3Pb_4I_{13}$/PTAA/ITO	one-step	12.23	45% ± 5%	720 h	0.8	[8]
Ag/PEI/PCBM/$(PEA)_2(MA)_4Pb_5I_{16}$/PTAA/ITO	one-step	11.1	60% ± 5%	500 h	0.7	[14]

**Au/Spiro-OMeTAD/$(PEA)_2(FA)_{n-1}Pb_nI_{3n+1}$/BI-$TiO_2$&$SnO_2$/FTO**	two-step	11.4	60%	900 h	0.86	[28]
**Ag/MoO_3/$(PEA)_2(Cs)_3Pb_4I_{13}$/TiO_2/FTO**	one-step	15.92	Nitrogen-fill	1440 h	0.9	[39]
**Ag/PEI/PCBM/$(PEA)_2(MA)_4Pb_5I_{16}$/PTAA/ITO**	one-step	11.1	50% ± 5%	800 h	0.65	[76]
**Ag/BCP/$PC_{61}BM$/$(PEA)_2(MA)_4Pb_5I_{16}$/PEDOT:PSS/ITO**	one-step	11.54	55% ± 5%	1440 h	0.88	[77]
**Ag/PCBM/$(PEA)_2(Cs)_3Pb_4I_{13}$/NiO/FTO**	one-step	9.84	15% ± 5%	720 h	0.7	[78]
**Au/Spiro-MeTAD/$(PEA)_2(MA)_4Pb_5I_{16}$/SnO_2/ITO**	two-step	13.0	70% ± 5%	720 h	0.81	This work
**Au/Spiro-OMeTAD/$(PEA)_2(MA)_4Pb_5I_{16}$/CsF–SnO_2/ITO**	two-step	14.5	70% ± 5%	720 h	0.87	This work
**Au/Spiro-OMeTAD/$(PEA)_2(MA)_4Pb_5I_{16}$/CsCl–SnO_2/ITO**	two-step	16.0	70% ± 5%	720 h	0.82	This work

Figure S1a indicated that the initial state of the dissolved PbI_2/PEAI precursor was initiated by the strong coordination of Pb^{2+} by the appropriate amount of DMSO and DMF from 0 to 140 min. Compared to the first priming containing pure DMSO, the first primer containing pure DMF immediately turned yellow during annealing at 70°C under atmospheric conditions, which further verified that the strong polarity of DMSO retarded the crystallization rate (Figure S1b). This experiment provided the solubility of different solvents in the precursor.

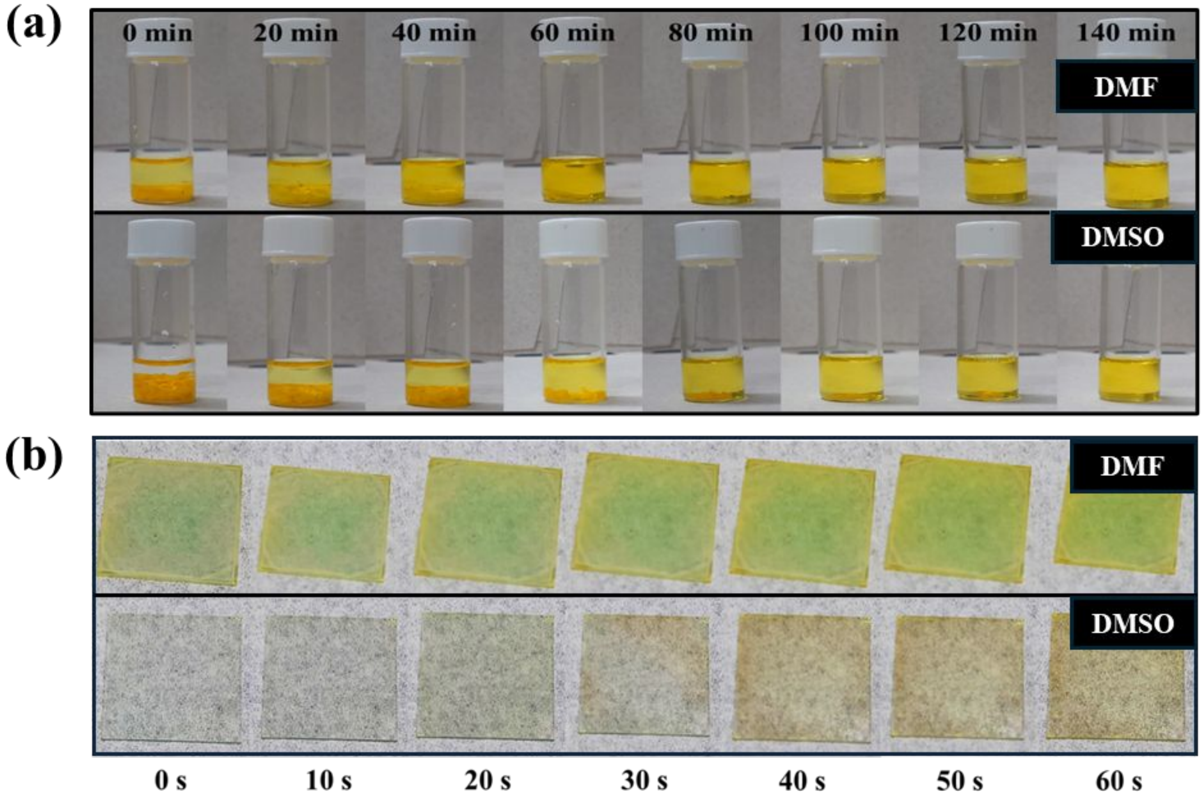

Figure S1. (a) Comparison of PbI_2/PEAI precursor dissolution speed in pure DMF and DMSO immersion solvents at room temperature. (b) Optical images of the first priming layer crystals grown on a hot plate at 70°C at different immersion durations in pure DMF and DMSO immersion solvents.

Figures S2a and S2b demonstrated the ITO substrate coverage by the two solvents at different ratios. The ITO substrate was treated with oxygen plasma before the deposition process. The best performance for 100% coverage of ITO/glass substrate was obtained for the first priming layer at DMF: DMSO = 10:0 and 8:2 and immersion time from 5 to 25 s. The first priming layer was obtained for 100% ITO/glass substrate coverage.

(a)

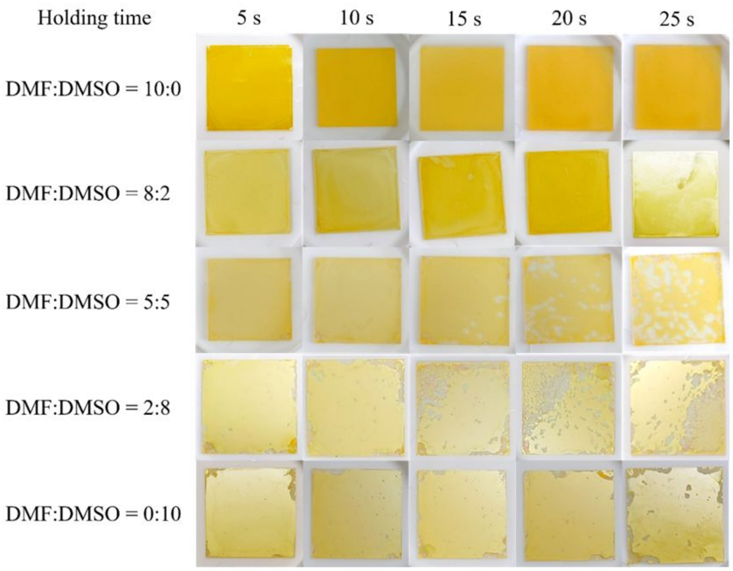

(b)

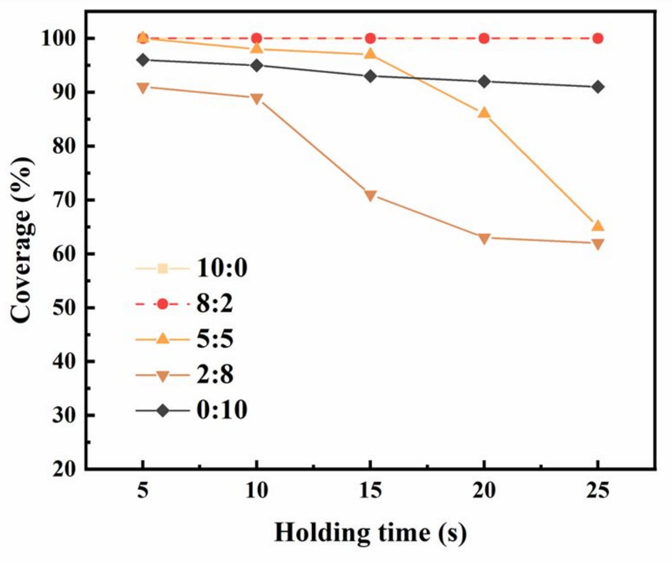

Figure S2. (a) Optical images of the first priming layer were obtained according to different

immersion times and DMF/DMSO ratios (after annealing for 10 min on a 70°C hot plate). (b) The calculated substrate coverage of the first priming layer (first step).

Next, the relevant investigation of the HIS-based first priming layer was carried out, and it was observed from the SEM with different magnifications that the films prepared in DMF: DMSO = 8:2 had a nanoporous morphology, which was favorable for the penetration and embedding of MA^+ in the second step of the process, as shown in Figure S3a. The UV-vis (Figure S3b) and XRD (Figure S3c) results also showed the prototype of Q-2D PF. Typical Q-2D film feature peaks were observed, similar to the Ruddlesden-Popper crystals feature. In order to obtain well-crystallized Q-2D PF that maintained good substrate coverage in subsequent processes, it was suggested to use HIS rather than a single solvent.

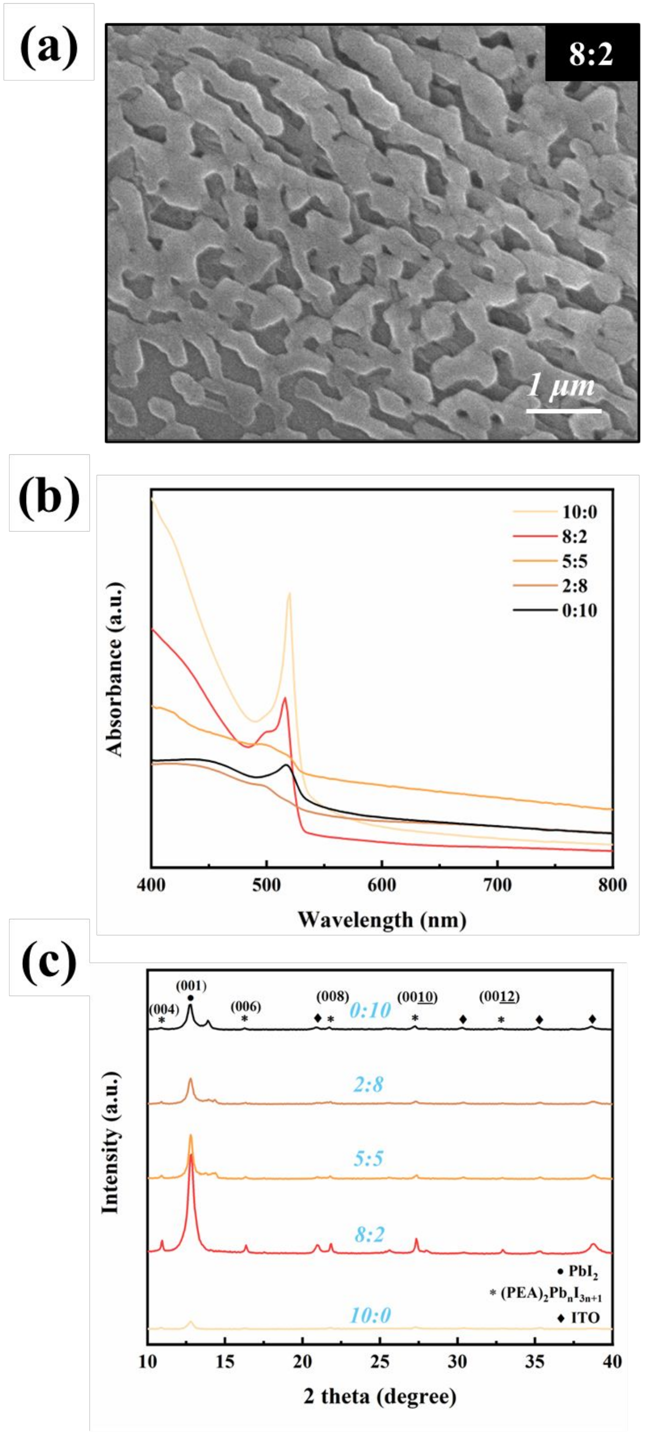

Figure S3. (a) SEM image at different magnifications of the first priming layer of the nanoplates with a nanoporous surface obtained in the first step (DMF/DMSO = 8:2). (b) UV−vis spectra and

(c) XRD pattern of the first priming layer obtained from the first step using HISs with different solvent ratios.

The grain sizes of the Q-2D PF prepared with different solvents at high humidity were displayed in Figures S4a and S4b. DMF/DMSO-based PFs had a much larger structure and higher grain size (8.39 μm) than DMSO-based PFs (4.86 μm). HIS system produced Q-2D PF with fewer grain boundaries and orientation stripes perpendicular to the boundaries. The HIS-based Q-2D PFs showed an optical bandgap of 1.8 eV, which indicateed the $n > 5$ phase (Figures S4c).

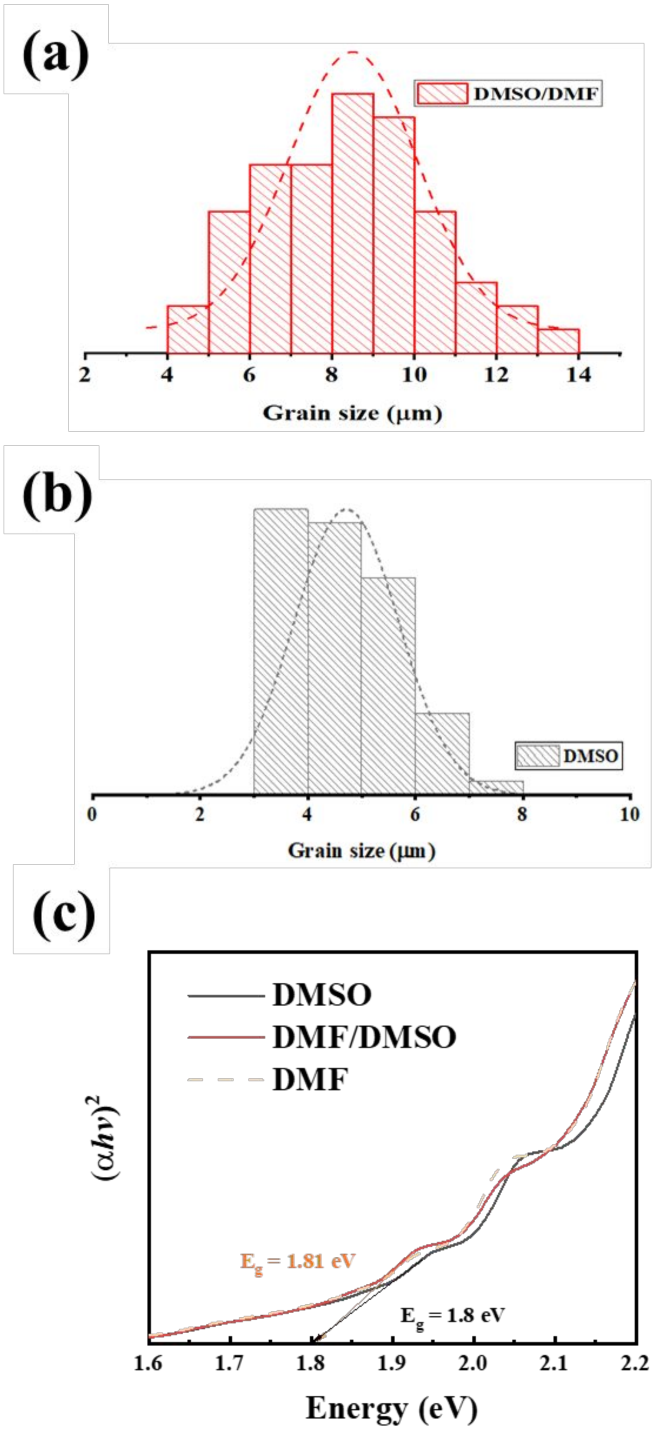

Figure S4. Q-2D $(PEA)_2MA_4Pb_5I_{16}$ film grain size distribution analysis prepared using (a) HIS and (b) DMSO-based first priming layer (MAI dropping amount of 110 μL). (c) Corresponding Tauc plot of Q-2D $(PEA)_2MA_4Pb_5I_{16}$ films based on HISs with different solvent ratios.

Figures S5 and S6 showed the Q-2D PFs obtained after the second step with various MAI amounts in different highly humidity environments. The Q-2D PMPI obtained by dropwise addition of 110-μL MAI had the best film quality. Clear grain boundaries remained at about 60% ± 5% relative humidity. It still had a clear stack and sharp grain boundaries, proving that the two-step process and HIS system contributed to the outstanding deposition of Q-2D PIPF in highly humidity environments. We can realize that the optimum amount of drop is 110 μL from the SEM, XRD and PL results.

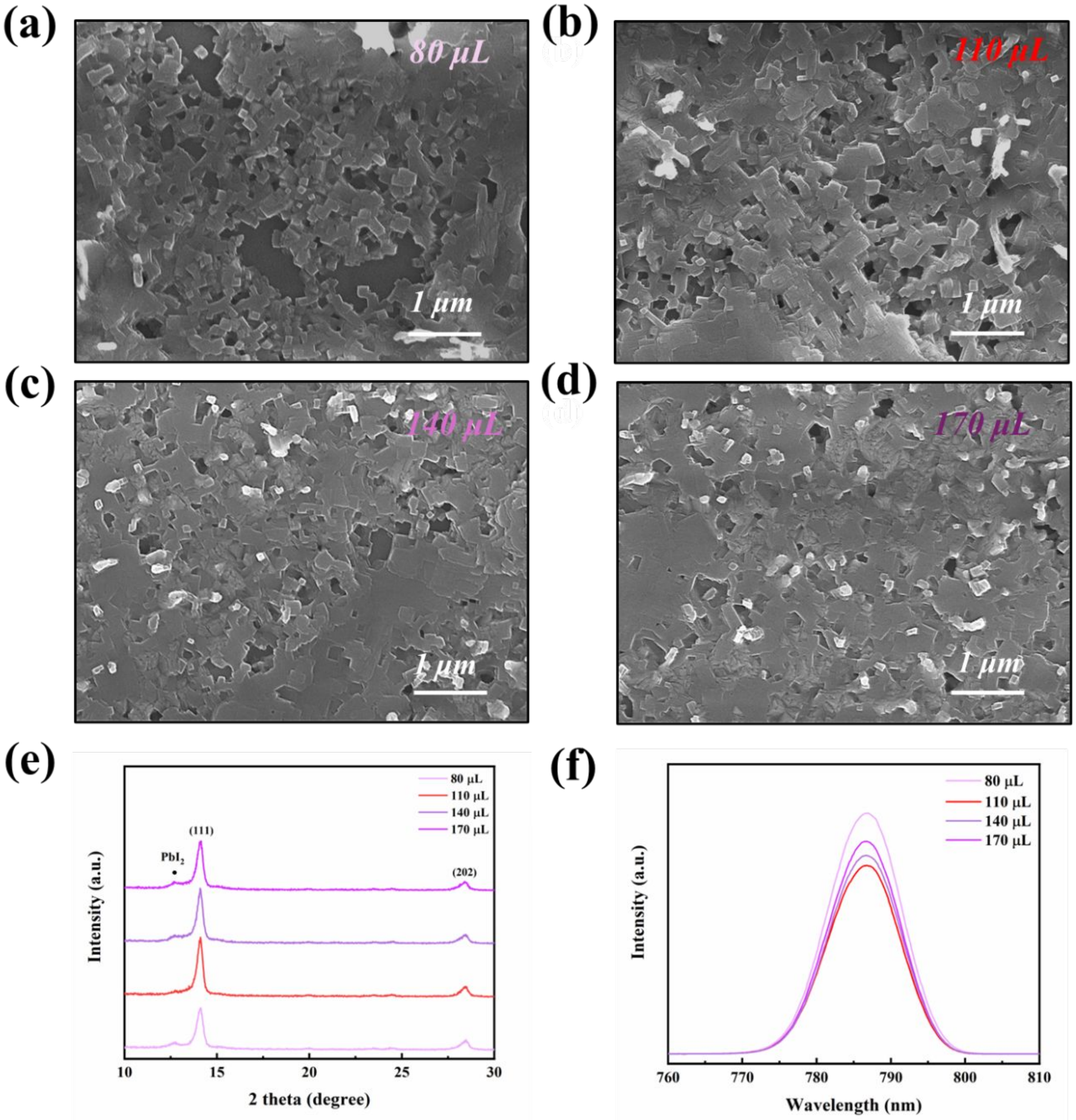

Figure S5. (a–d) Top views of SEM images of the standard Q-2D structure. (e) XRD pattern and (f) PL spectra of square-shaped grains of Q-2D $(PEA)_2MA_4Pb_5I_{16}$ films with different MAI dropped amounts in the second step.

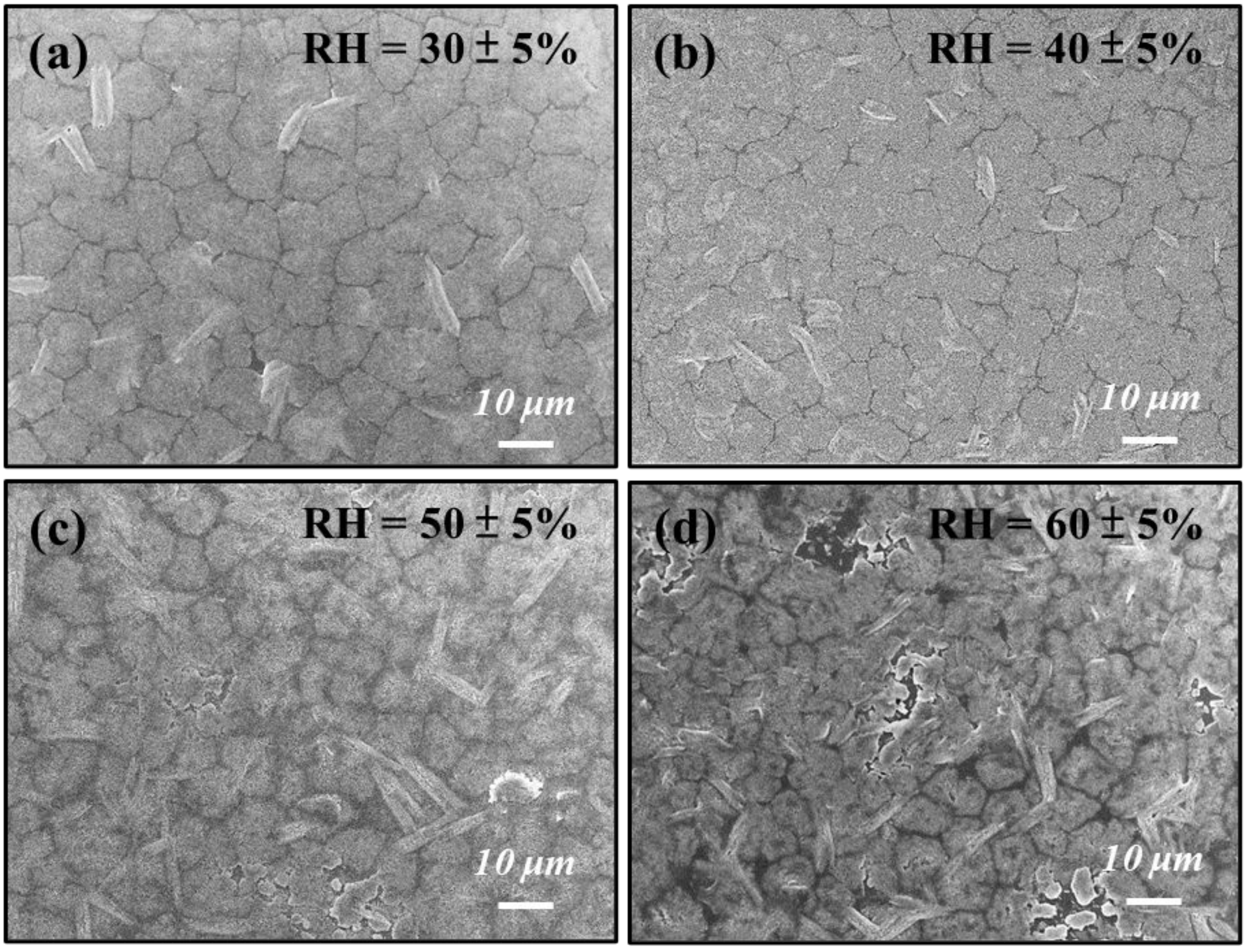

Figure S6. SEM images of Q-2D $(PEA)_2MA_4Pb_5I_{16}$ fabricated in different relative humidities in the second step: (a) 30% ± 5%, (b) 40% ± 5%, (c) 50% ± 5%, and (d) 60% ± 5% RHs.

As shown in Figure S7, the XPS pattern was carried out to track the signal of the chemical states of the surface of Q-2D PIPF. All XPS spectra were calibrated using C 1s spectra. All the peaks belong to the perovskite phase, and no peaks of metallic Pb were observed in the spectra, realizing the complete conversion of the two-step preparation of Q-2D PIPF.

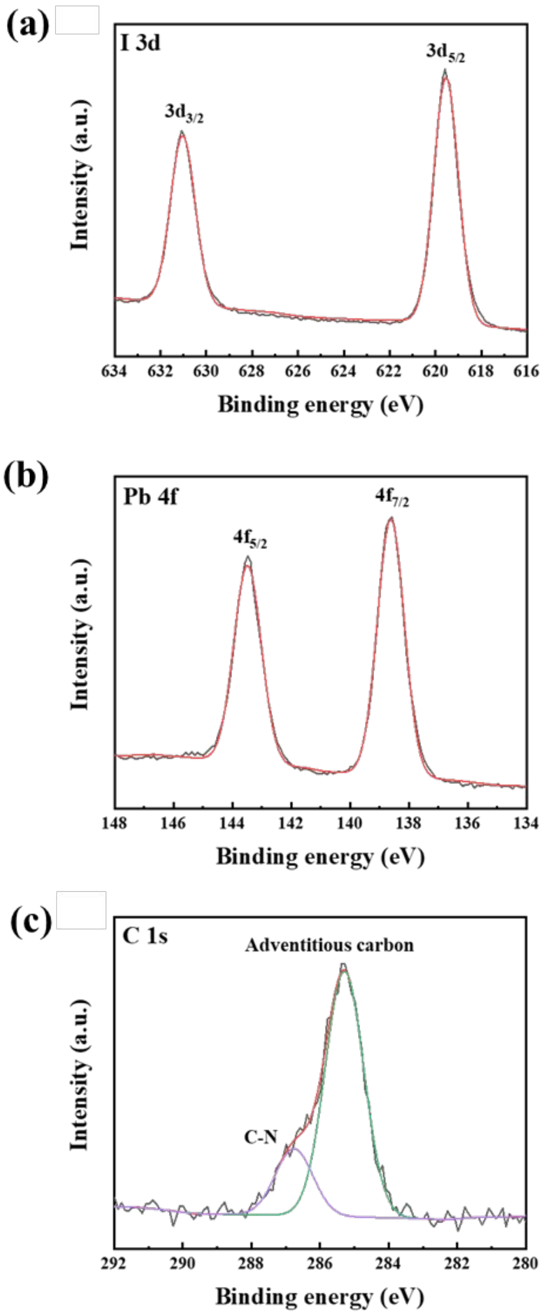

Figure S7. XPS spectra of (a) I 3d, (b) C 1s, and (c) Pb 4f of Q-2D $(PEA)_2MA_4Pb_5I_{16}$.

Figures S8 and S9 compared the changes in grain size of SEM images of ETL with different modification concentrations. When the modified concentration of CsF and CsCl was increased, contact angle images (inset) indicated that the surface hydrophilicity was better, which was more

suitable for depositing perovskite on the upper layer. The average grain size also increased slightly as the concentration increases (Figure S8f). The increase in ETL thickness also benefited from the modification of CsF and CsCl.

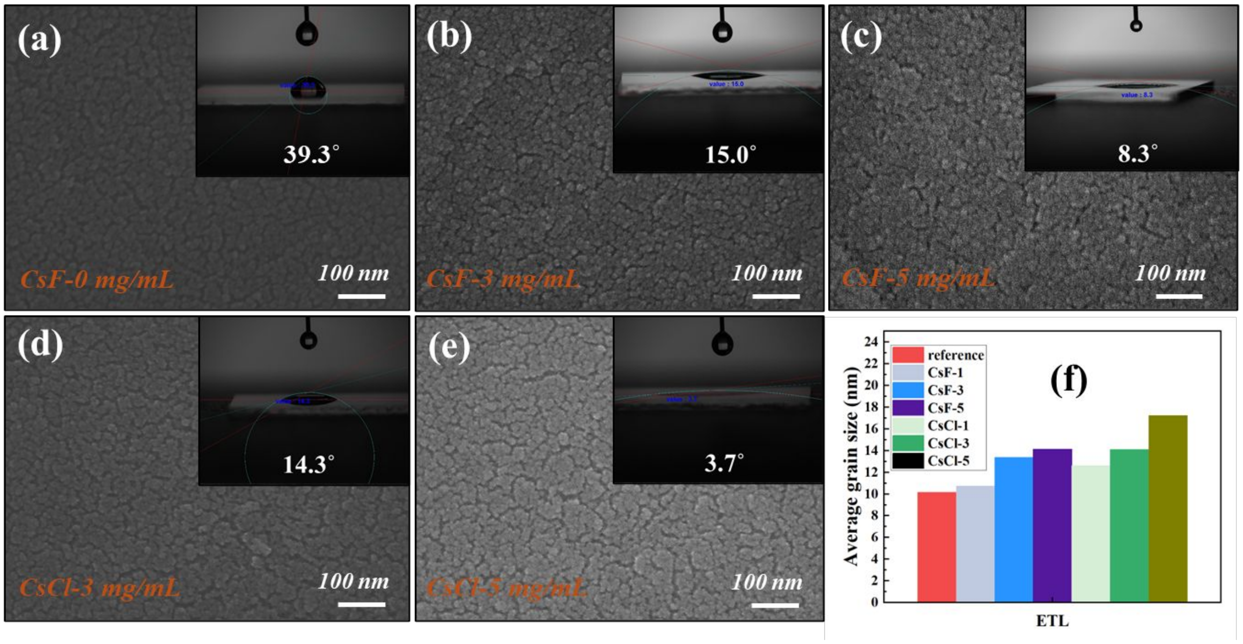

Figure S8. SEM top-view image of SnO_2-based ETLs with (a) CsF-0, (b) CsF-3, (c) CsF-5, (d) CsCl-3, and (e) CsCl-5 mg mL^{-1}. The inset shows contact angle measurement. (f) The average grain size of various ETLs.

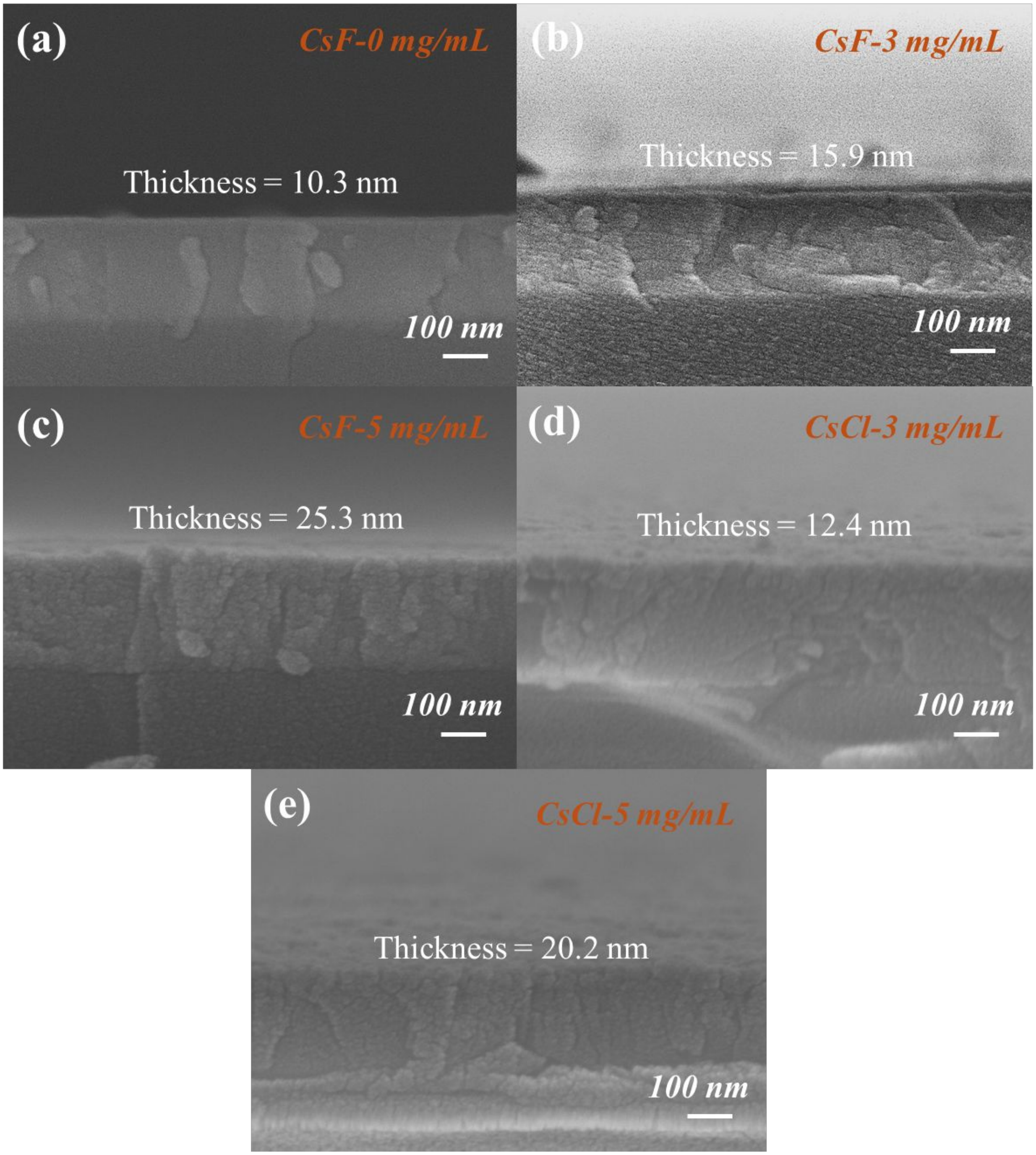

Figure S9. SEM cross-section images and thickness of SnO_2-based ETLs modified with different ionic solutions. (a) Pristine SnO_2, (b) CsF-3, (c) CsF-5, (d) CsCl-3, and (e) CsCl-5 mg mL^{-1}.

In order to further investigate the innate aggregation phenomenon in SnO_2 colloidal solutions, a DLS analysis and zeta potential measurement were illustrated in Figures S10 and S11. It was confirmed that there was no excessive aggregation of SnO_2 after modification and that the average grain size, although growing with the increase in concentration, was still within the appropriate range. Interestingly, the grain size grew with increasing modification concentration in colloidal solution, and the results were similar to its thickness observation in Figure S9. Zeta potential results were also consistent with Table S2.

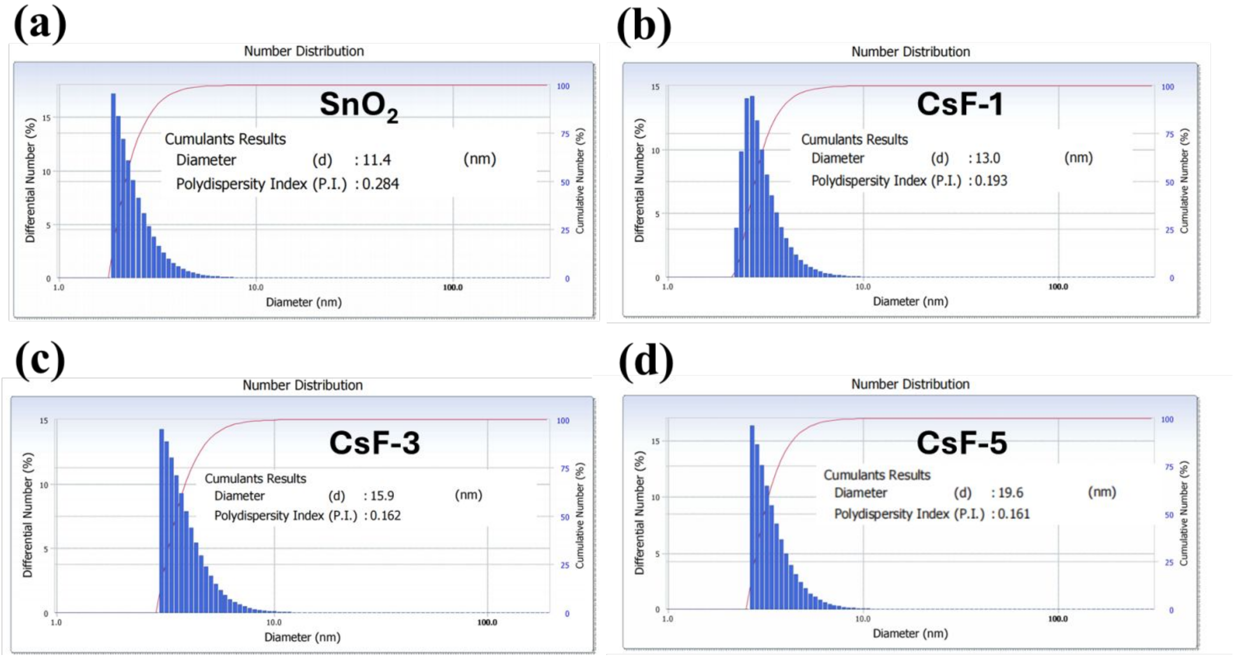

Figure S10. DLS number–average distributions and cumulative proportion of different sizes of SnO_2 particles in (a) 0, (b) 1, (c) 3, and (d) 5 mg mL^{-1} of the CsF solution.

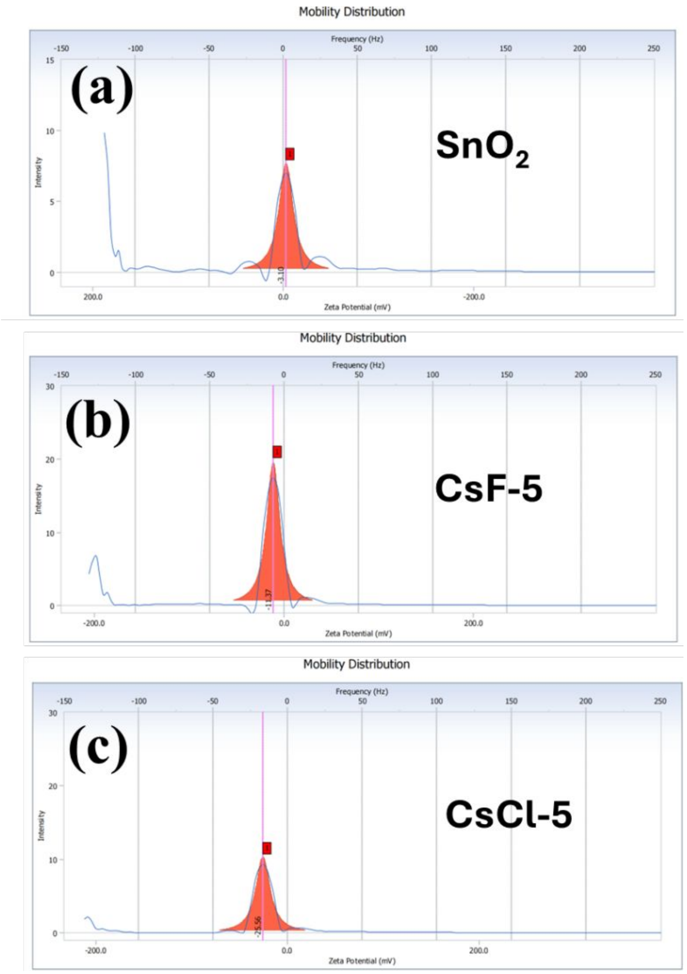

Figure S11. Zeta potential of (a) SnO_2, (b) CsF-5, and (c) CsCl-5 mg mL^{-1} doping concentrations.

Following the previous confirmation that no aggregation of the SnO_2 colloidal solution occurred even after 7 days of placement, the dispersion of the additive in the SnO_2 film after annealing was verified in Figure S12. The EDS results showed that the ETL was uniformly modified after annealing, and the additives were uniformly dispersed in the SnO_2 film without aggregation.

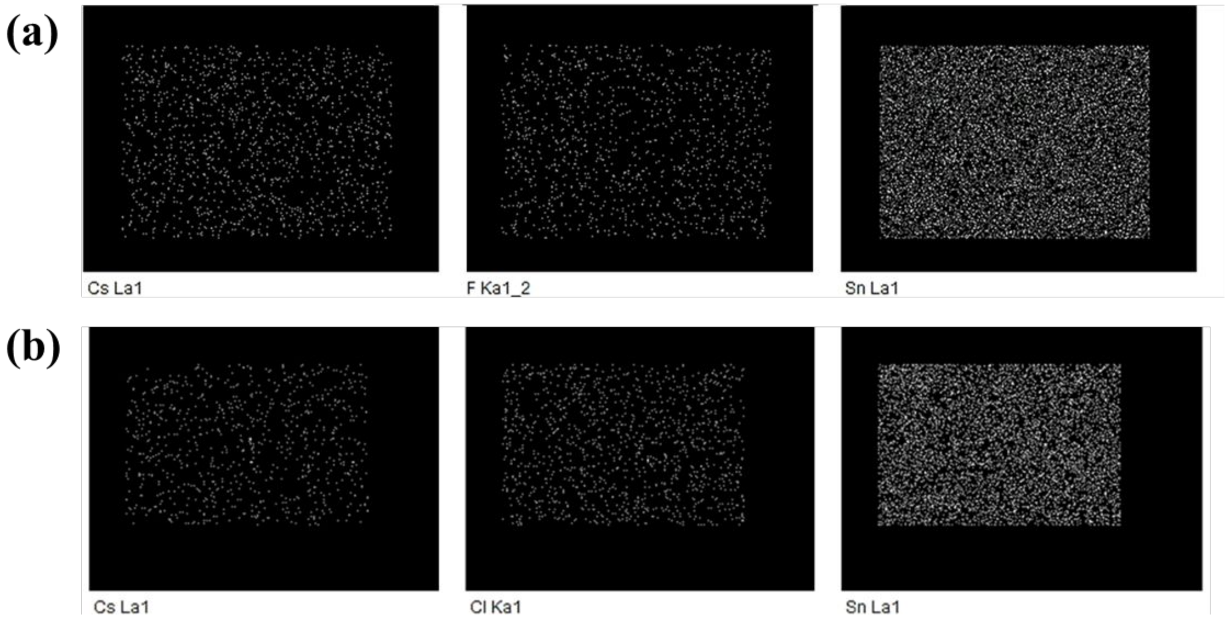

Figure S12. Elemental mapping of EDS of (a) CsF-3- and (b) CsCl-3-modified ETLs.

We compared the morphology of Q-2D PMPI deposited on CsF-3-SnO_2 and CsCl-3-SnO_2 ETLs in Figures S13a and S13b. The Q-2D PMPIs formed dense and continuous thin films, which contributed to the better hydrophilicity of the modified ETLs (Figure S8), and also resulted in a larger average grain size than that of the Q-2D PMPI films on unmodified SnO_2.

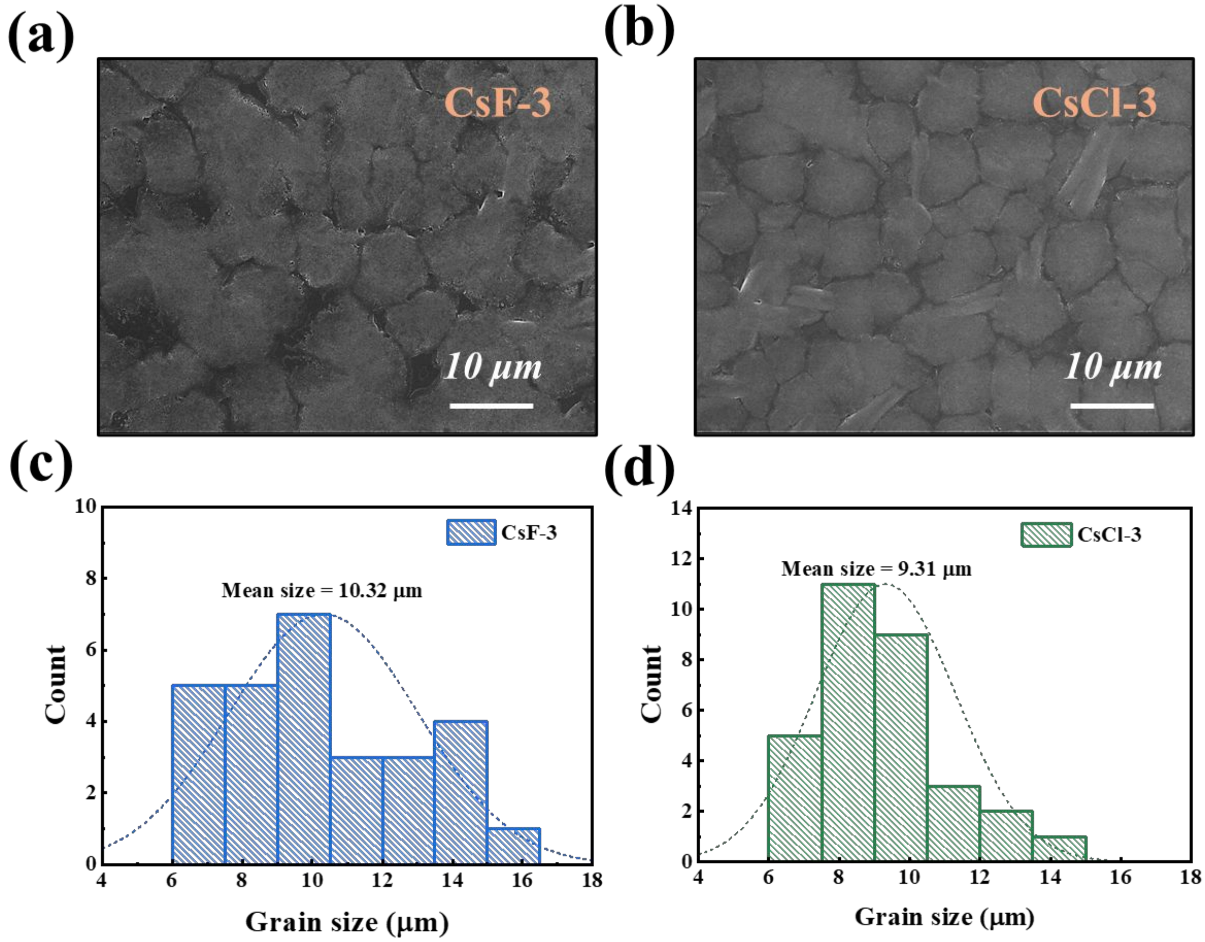

Figure S13. (a and b) SEM morphology and (c and d) grain size distribution analysis of Q-2D $(PEA)_2MA_4Pb_5I_{16}$ films deposited on CsF–SnO_2 and CsCl–SnO_2 ETLs.

While the optimization of the perovskite and ETL layers, the HTL in Figure S14 still deteriorated over time and was also responsible for the decrease in PCE.

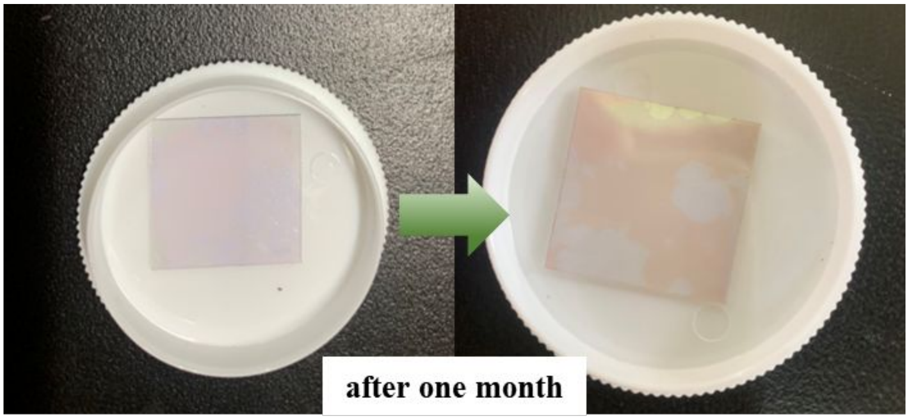

Figure S14. Optical images of HTL after aging in the air after 1 m.
